# Powassan Virus in the United States: An AI-Integrative One Health Scoping Review of Ecological Drivers, Zoonotic Interactions, Symptom Profiles, and Public Health Implications

**DOI:** 10.3390/idr18050096

**Published:** 2026-08-31

**Authors:** Sarah P. Maxwell, Connie L. McNeely, James R. Harrington, Kevin C. Thomas

**Affiliations:** 1 School of Economic, Political, and Policy Sciences, University of Texas at Dallas, Richardson, TX 75080, USA; 2 Schar School of Policy and Government, George Mason University, Fairfax, VA 22030, USA; 3 Laboratory for Human Neurobiology, Boston University School of Medicine, Boston, MA 02118, USA

**Keywords:** Powassan virus, One Health, zoonosis, flavivirus, *Ixodes scapularis*, tick-borne disease

## Abstract

Background: Powassan virus (POWV) is the only tick-borne encephalitis-group flavivirus endemic to the United States (U.S.) and is an increasingly serious public health threat. Reported U.S. cases remain relatively few but have grown steadily—from fewer than two annually before 2005 to a record 60 in 2024—and neuroinvasive disease carries a 10–15% case fatality with lasting neurological sequelae in roughly half of the survivors. Examining POWV through a One Health lens, we conducted a scoping review of its ecological and zoonotic drivers, its interactions with co-circulating tick-borne pathogens, its full clinical spectrum, and its distinction from other tick-borne diseases. Methods: Following the Preferred Reporting Items for Systematic Reviews and Meta-Analyses extension for Scoping Reviews (PRISMA-ScR) guidance, we searched PubMed/MEDLINE, U.S. Centers for Disease Control and Prevention (CDC) surveillance databases, state health-department publications, and vetted public-health news through early 2026. The review was conducted as an AI-integrative partnership among the authors, the Global Infectious Diseases and Epidemiology Online Network (GIDEON) database, and Claude (Anthropic), enabling a synthesis of human-curated archival records collected over six years alongside the published literature; this yielded over 200 sources, almost half unavailable through an AI search alone. Results: POWV is maintained in a sylvatic cycle involving *I. scapularis* ticks, small-mammal reservoirs, and incidental human spillover. Its clinical spectrum spans asymptomatic seroconversion to fatal meningoencephalitis; prodromal fever, headache, vomiting, and fatigue may progress to encephalitis, seizures, aphasia, cranial-nerve palsies, paralysis, and ataxia—the most severe neurological syndrome among U.S. tick-borne diseases. Cases cluster in the northeastern and Great Lakes states, with older males disproportionately affected, while viral evidence extends to the mid-Atlantic, southern, and western regions. POWV is distinguished by its flaviviral etiology, short (as little as 15 min) transmission window, absence of a characteristic rash, and complete antibiotic resistance. Of the 48 states surveyed, only 7 published POWV data. Conclusions: POWV is an escalating One Health threat driven by expanding vector tick ranges, land-use and climate change, and wildlife–human interface dynamics. Enhanced cross-sector surveillance integrating human, animal, and environmental monitoring, alongside improved public-health data reporting, is urgently needed.

## 1. Introduction

Powassan virus (POWV) is a positive-sense, single-stranded RNA arbovirus classified within the family *Flaviviridae*, genus *Flavivirus* [1]. It belongs to the tick-borne encephalitis (TBE) serocomplex alongside TBE virus, Omsk hemorrhagic fever virus, and Kyasanur Forest disease virus [2]. POWV holds a unique distinction as the only tick-borne flavivirus endemic to North America [3]. From a One Health perspective—which recognizes the inextricable interdependence of human, animal, and environmental health—POWV is the causative agent of a paradigmatic zoonosis, i.e., an infectious disease that is transmitted between vertebrate animals and humans [4]. Its emergence and geographic expansion are products of ecological disruption, climate change, wildlife population dynamics, and expanding human activity in natural habitats, making it impossible to understand POWV risk in humans without simultaneously examining the virus in its broader ecological context [5].

**Figure 1:**
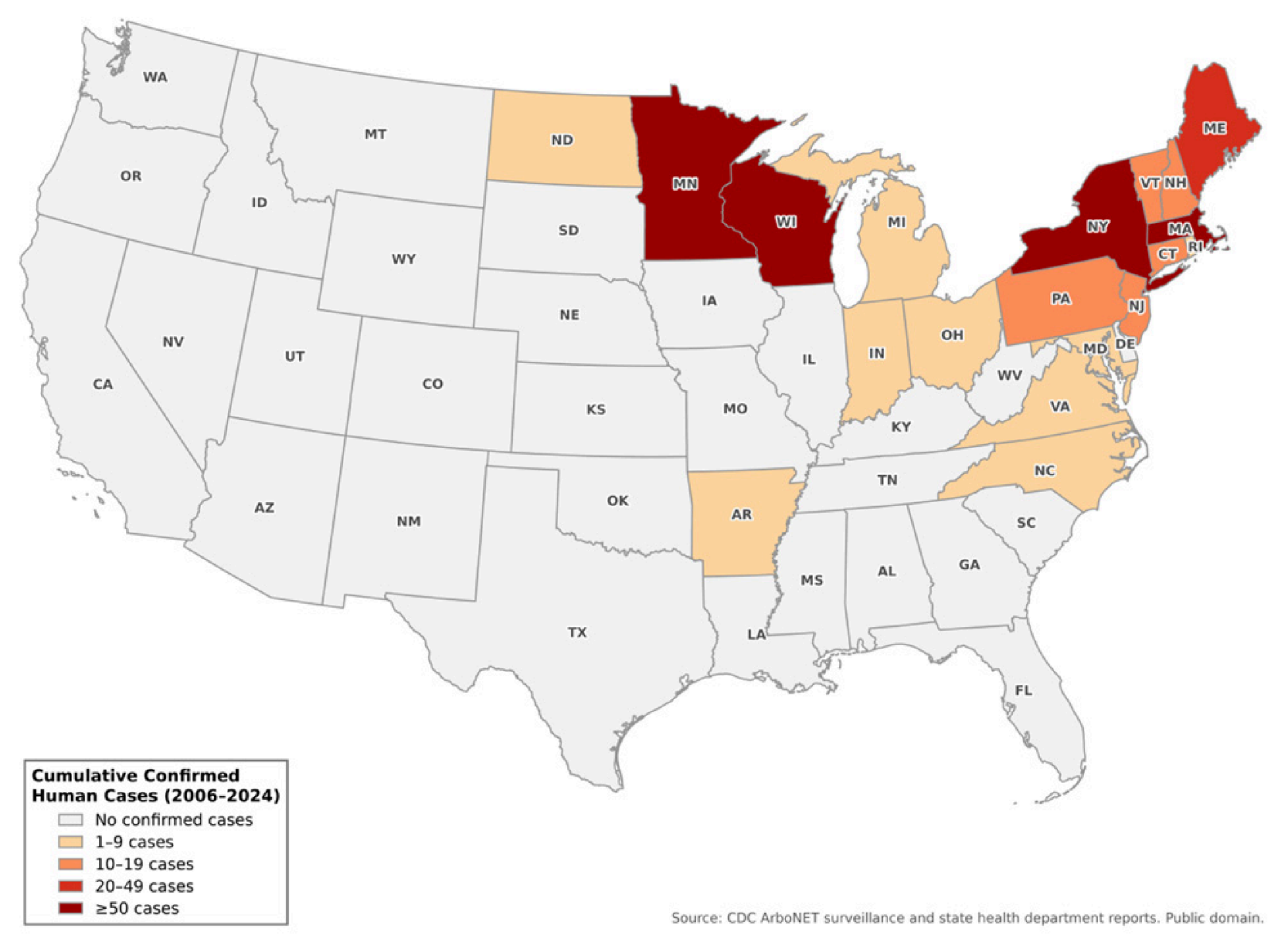
Geographic distribution of reported Powassan virus disease cases in the United States, 2006–2024. Map data are sourced from CDC ArboNET surveillance [11]. For interactive map access, see: https://www.cdc.gov/powassan/data-maps/historic-data.html (accessed on 3 June 2026) [11]. Additional peer-reviewed maps depicting POWV case distributions and tick range overlap are available in Hassett & Thangamani [12], Vogels et al. [13], and Kakoullis et al. [14].

The virus was first isolated in 1958 from a fatal pediatric encephalitis case in Powassan, Ontario, Canada [6]. Retrospective analyses subsequently recovered the virus from *Dermacentor andersoni* ticks in Colorado as early as 1952, indicating cryptic circulation predating its human characterization [7]. Between 1958 and 1998, only 27 human cases were documented across eastern Canada and the northeastern United States—fewer than one per year [8]. The epidemiological landscape changed markedly in the 2000s: nine confirmed cases were reported in the U.S. between 1999 and 2005 alone, including the first cases from Michigan and Wisconsin [8]. Reported cases accelerated through the following two decades, totaling 99 cases in 2006–2016, and a record 330 cases in 2014–2024 [9,10,11], and are increasing. The geographic distribution of reported cases is illustrated in Figure 1.

The drivers of this epidemiological shift are explicitly ecological in nature, underscoring the One Health relationship. The geographic range of the primary vector, *Ixodes scapularis* (black-legged tick), has expanded substantially northward and westward over the past four decades, driven by climate warming, reforestation, tick-carrying white-tailed deer population growth, and suburban residential land-use change—new housing development and the roads and utility infrastructure that accompany it at the urban–forest interface—that increases tick–wildlife–human contact [12,15]. Rising tick densities in peri-urban environments, changing recreational and occupational patterns bringing humans into tick habitats, and the amplification of sylvatic transmission cycles among diverse mammalian reservoir hosts collectively create conditions conducive to POWV spillover into human populations [15,16]. In parallel, improved diagnostic testing and heightened clinician awareness have increased case ascertainment, so that part of the rise in reported incidence reflects better detection superimposed on genuine ecological expansion [17,18].

Despite its increasing incidence, POWV remains substantially underrecognized. Seroprevalence data from endemic states estimate a 0.7–6.1% population-level antibody prevalence—vastly exceeding reported clinical case counts—suggesting that most POWV infections are asymptomatic or cause only non-specific febrile illness [19,20]. This surveillance gap is compounded by low clinician familiarity, limited availability of POWV-specific diagnostic testing outside state public health laboratories, and the virus’s mimicry of other encephalitides [18]. For those who do develop neuroinvasive disease, POWV is devastating: 10–15% of neuroinvasive cases are fatal, and approximately 50% of survivors sustain lasting neurological deficits [18,21]. Crucially, unlike most tick-borne pathogens that require hours of tick attachment for transmission, POWV can be transmitted in as little as 15 min, rendering standard post-bite tick removal strategies insufficient for prevention [22].

Offering a scoping review, this study synthesizes the published clinical, epidemiological, and public health literature on POWV in the United States with a dual focus: characterizing POWV’s clinical symptom profile and situating that profile within its One Health ecological context. Understanding POWV requires acknowledging that the patient who presents to a clinician with encephalitis represents the end product of a chain of events that likely began in a forest, proceeded through tick–wildlife interaction, was modulated by land use and climate, and culminated in a human disease encounter [23]. Addressing POWV comprehensively—in surveillance, prevention, and ultimately in treatment—requires integrating human medicine, veterinary science, ecology, and environmental health policy. This review was produced through a novel AI-integrative partnership among the primary human investigators, the GIDEON Infectious Diseases Database, and Claude (Anthropic). This approach—in which a human expert introduces curated, archival, and database-sourced materials to an AI synthesis partner—is described in detail in the Methods section and represents a transparent model for human–AI collaboration in biomedical evidence synthesis.

Seven research questions guided this research, organized within a One Health framework that positions human disease as the downstream output of animal, ecological, and environmental systems: (1) What ecological, environmental, and zoonotic factors drive the transmission and geographic expansion of POWV in the United States, and how do changes in wildlife communities, tick vector populations, land use, and climate shape human exposure risk? (2) How does POWV interact with co-circulating tick-borne pathogens—including *Borrelia burgdorferi*, *Anaplasma phagocytophilum*, *Babesia microti*, and others—within shared tick vectors and wildlife reservoir communities, and what are the implications of coinfection for clinical recognition and disease burden? (3) What is the role of wildlife sentinel surveillance—particularly white-tailed deer and small mammal reservoir seroprevalence—as an early-warning indicator of increasing human POWV exposure risk? (4) What is the full spectrum of clinical symptoms associated with POWV infection in the United States, and how do symptom profiles relate to host ecological exposure pathways? (5) How do POWV symptoms and outcomes vary by geographic location, and what ecological characteristics of those locations explain the observed patterns? (6) How do POWV symptoms and clinical outcomes differ by patient age and sex, and what biological and behavioral exposure factors rooted in human–environment interaction contribute to those differences? (7) How do POWV symptoms differ from those of other tick-borne diseases that share vectors, reservoir hosts, and geographic range, and what are the implications of that shared ecology for integrated surveillance and differential diagnosis?

## 2. Background: Virology, Vectors, and the One Health Transmission Cycle

### 2.1. Tick Vectors, Reservoir Hosts, and the Sylvatic Cycle

**Figure 2:**
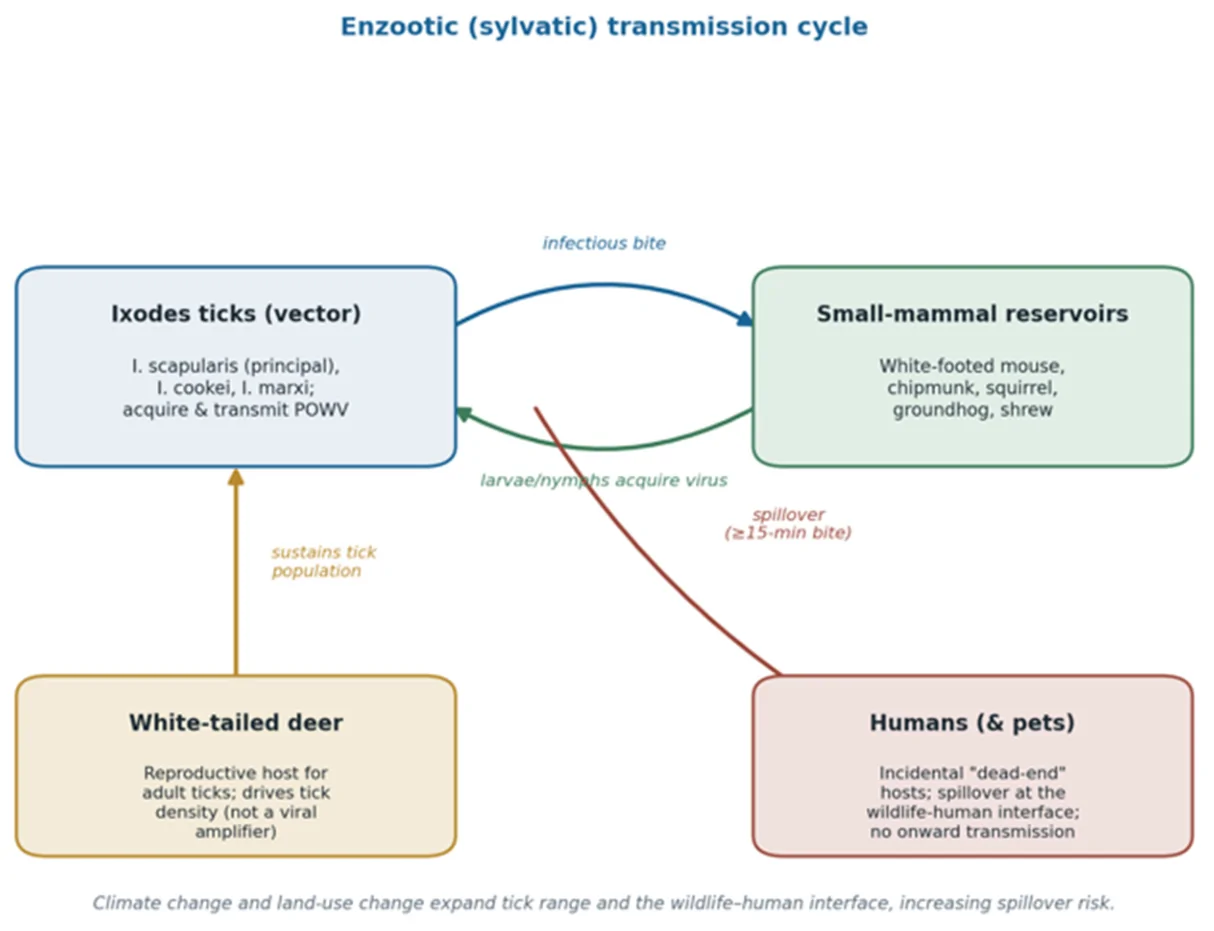
Enzootic (sylvatic) transmission cycle of the Powassan virus. POWV is maintained between *Ixodes* ticks and small-mammal reservoir hosts; white-tailed deer sustain the tick population without amplifying the virus, and humans (and pets) are incidental dead-end hosts infected by spillover at the wildlife–human interface, where a bite of as little as 15 min can transmit the virus. Climate and land-use change expand tick range and the wildlife–human interface, increasing spillover risk.

From a One Health perspective, POWV transmission is embedded within a complex sylvatic cycle involving multiple tick species and diverse mammalian hosts (Figure 2). Three *Ixodes tick* species serve as recognized vectors in North America [12]. *I. scapularis* is the principal vector of deer tick virus (DTV; POWV lineage II), the tick most responsible for human infections in the eastern United States [24]. *I. cookei* (the groundhog tick) is the primary lineage I vector but rarely bites humans due to its host preference for medium-sized burrowing mammals [25]. *I. marxi* (the squirrel tick) carries lineage I virus but also has limited human contact [26]. Importantly, *Dermacentor andersoni,* the Rocky Mountain wood tick, was the species from which POWV was first isolated in Colorado in 1952, indicating that the virus utilizes a broader vector range under certain ecological conditions [7]. Notably, POWV circulation is neither restricted to North America nor confined to ticks: in the Russian Far East (Primorsky Krai), where POWV is also endemic, the virus has been detected in mosquitoes in addition to ixodid ticks, indicating that under some ecological conditions, its arthropod host range extends beyond ticks [2,27].

Reservoir hosts include white-footed mice (*Peromyscus leucopus*), chipmunks, squirrels, groundhogs, and shrews [7,28]. White-tailed deer (*Odocoileus virginianus*), while not competent viral amplifiers, are the primary host for adult *I. scapularis* and function as critical drivers of tick population density [29]. Deer seroprevalence for POWV in Connecticut rose from under 25% before 1996 to 80–91% between 2005 and 2009—a dramatic indicator of increasing viral circulation in the wildlife reservoir [1]. This One Health signal in the deer population preceded and predicted the subsequent rise in human cases, underscoring the value of animal sentinel surveillance as an early-warning system for human disease risk [19]. The geographic overlap among *I. scapularis* habitat, wildlife reservoir communities, and human POWV cases is depicted in Figure 1 and discussed in Section 5.

Uniquely among *I. scapularis*-borne pathogens, POWV can be transmitted within as little as 15 min of attachment—versus 36–48 h for *Borrelia burgdorferi*—so conventional tick-check-and-removal offers only limited protection and must be supplemented with repellents and protective clothing [22,30].

### 2.2. Climate Change, Land Use, and One Health Drivers of POWV Emergence

The rising incidence of POWV is inseparable from its ecological and environmental determinants—a prototypical One Health problem [4]. *I. scapularis* tick populations have expanded northward by approximately 46 km per decade over the past 20 years, driven by warming winters, reduced snow cover, earlier spring warming, and longer frost-free seasons [31]. Phylodynamic analyses of the DTV lineage confirm that viral population expansion has been concordant with *I. scapularis* range expansion over the past 50 years [31]. Climate projections anticipate continued northward and westward displacement of tick habitat, which will expose previously non-endemic human populations to POWV risk [16]. As with Lyme disease—whose U.S. incidence has risen sharply over the same period—climate change has very likely contributed to the increasing incidence of POWV disease by expanding the range, abundance, and seasonal activity of its *I. scapularis* vector [31,32].

At the scale of individual tick behavior, these climatic shifts operate through well-defined physiological mechanisms. *I. scapularis* is highly sensitive to temperature and to saturation deficit (the drying power of the air): questing activity—the host-seeking behavior that brings ticks into contact with wildlife and humans—rises as spring and autumn temperatures climb above roughly 4–7 °C, while sustained heat and low humidity force ticks to retreat into the leaf litter to rehydrate, limiting the daily window for host contact. Warmer winters and reduced snow cover improve the overwintering survival of nymphs and adults, longer frost-free seasons lengthen the questing season, and the high ambient humidity of closed-canopy, leaf-litter-rich microhabitats sustains the moisture that ticks require—which is why forest-edge and understory environments support the highest tick densities [31,33]. Because nymphal questing phenology governs when infected ticks are most likely to encounter people, these temperature- and humidity-dependent behaviors translate broad climate trends directly into the seasonality and geographic expansion of human POWV risk [31].

Land use changes compound climate drivers. Reforestation of formerly agricultural land in the northeastern United States has created extensive forest-edge habitat optimal for tick questing behavior and white-footed mouse abundance—both key elements of the POWV transmission cycle [12]. Suburban and exurban residential development at the urban–forest interface increases tick–human contact by bringing residents into habitats with established POWV enzootic cycles [17]. From a One Health policy perspective, land use planning, wildlife management, and residential tick control represent underutilized levers for POWV prevention at the population level [15,26,34].

Human demographic factors are equally important One Health drivers of POWV’s multi-state emergence. The northeastern United States—the historical core of POWV activity—has undergone a century-long transition from an agricultural to a post-industrial economy: widespread abandonment of farmland during and after industrialization, followed by the later decline of manufacturing, allowed for the extensive reforestation of former fields into the forest-edge habitat that now sustains dense tick and small-mammal communities [12,15]. Superimposed on this land-use legacy, human migration has redistributed populations out of urban centers and into suburban and exurban developments at the forest interface, placing growing numbers of residents in direct contact with enzootic transmission foci [15,17]. This in-migration, together with expanding outdoor recreational and occupational activity, has widened the human–tick interface across an increasing number of states and helps explain why recognized POWV disease has extended beyond its traditional epicenters into the mid-Atlantic, southern, and interior regions [16,17]. Human mobility also shapes the apparent multi-state distribution of disease directly: travel-associated infections—such as Maryland’s first confirmed case, acquired in Canada—show that human movement can separate the place of exposure from the place of diagnosis and reporting [35,36]. Accounting for these human factors—historical industrialization and the land-use transitions it set in motion, population migration toward the wildland–urban interface, and contemporary travel—is therefore essential to a complete One Health explanation of POWV emergence across the several states now reporting disease [5,37].

### 2.3. Pathogenesis and Neurotropism

Following tick bite, POWV replicates locally and disseminates hematogenously before crossing the blood-brain barrier to establish CNS infection [38]. The virus exhibits pronounced neurotropism, preferentially infecting neurons in the cerebral cortex, thalamus, basal ganglia, brainstem, cerebellum, and spinal cord [39]. Neuropathological examination reveals CNS edema, leukocyte infiltration, neuronal loss, gliosis, and tissue necrosis [40]. Age-dependent differences in neuroinflammatory cytokine profiles—specifically differential glial cell activation between young and old animals—explain in part the greater lethality of POWV in older hosts, a biological mechanism with direct relevance to clinical risk stratification [41]. Chronic neuroinflammation and persistent viral RNA, rather than ongoing active replication, appear to underlie long-term sequelae in survivors [42].

### 2.4. One Health Ecological Interactions: POWV, Wildlife, and Co-Circulating Zoonotic Pathogens

Powassan virus exists not as an isolated pathogen but as one element of a richly interconnected zoonotic disease system anchored by *Ixodes scapularis* ticks and the shared ecological community they parasitize. Understanding POWV from a One Health perspective requires examining its relationships with the wildlife communities that maintain it, the environmental forces that shape those communities, and the co-circulating tick-borne pathogens that share its vector, its reservoir hosts, and—increasingly—its human patients [4,5].

The enzootic maintenance cycle depends on a small-mammal reservoir community—principally the white-footed mouse (*Peromyscus leucopus*), together with chipmunks, squirrels, shrews, and groundhogs—that bridges infected and naïve tick cohorts, with nymphal *I. scapularis* responsible for most human infections [7,12,28]. White-tailed deer (*Odocoileus virginianus*) are not viral amplifiers but, as the primary reproductive host for adult ticks, are the principal driver of tick population size; their expansion across the northeastern and upper-midwestern United States has been a central ecological engine of *I. scapularis* range expansion and POWV emergence [12,15,29]. Serial deer serosurveys compiled in GIDEON reinforce this trajectory, with regional POWV seroprevalence rising from 4% in 1979 to 91% by 2009—among the clearest wildlife-sentinel signals of intensifying enzootic circulation in the Northeast [1,43,44].

Beyond deer, shrews are competent reservoir amplifiers, and the prototype (lineage I) virus—vectored chiefly by *Ixodes cookei*—has been recovered from *Ix. spinipalpis*, *Ix. marxi*, and *Dermacentor andersoni*, while *Ix. scapularis* transmits the lineage II deer tick virus; POWV was detected in a Delaware tick for the first time in March 2026, extending the documented vector range along the mid-Atlantic [43,45,46]. POWV-reactive antibodies have been reported in white-tailed deer, snowshoe hares, and other mammals across North America—including states with no recognized human cases—confirming that the enzootic footprint substantially exceeds the documented human case range [19,27,47,48].

POWV shares its *I. scapularis* vector and small-mammal reservoirs with a suite of co-circulating human pathogens, whose relationships with POWV bear directly on clinical recognition and surveillance. The complete set of human pathogens transmitted by *I. scapularis* in the United States comprises *Borrelia burgdorferi* and *Borrelia mayonii* (Lyme disease), *Borrelia miyamotoi* (hard-tick relapsing fever), *Anaplasma phagocytophilum* (anaplasmosis), *Ehrlichia muris eauclairensis* (ehrlichiosis), *Babesia microti* (babesiosis), and POWV/deer tick virus [49]. Because these agents share the white-footed mouse reservoir and the same larval and nymphal tick stages, individual ticks in highly endemic areas carry two or more of them at coinfection rates of 10–25%, and a single bite may deliver several simultaneously [50,51,52,53,54]. Tick-based surveys aggregated in GIDEON detected deer tick virus in 0.9% of *Babesia microti*-positive *Ixodes* ticks in Pennsylvania (2021–2023) and in 0.6–1.72% of *I. scapularis* across New Jersey and Pennsylvania (2018–2021) [50,51,52]. The clinical significance of POWV coinfection is not fully characterized, but this ecological co-occurrence means that a patient with tick-borne illness may harbor multiple simultaneous infections—a complexity better recognized by an ecologically informed One Health approach than by a single-pathogen framework [49,55].

Microbiome analyses of *I. scapularis* tick pools from New York and Connecticut have confirmed simultaneous carriage of POWV, *B. burgdorferi*, *A. phagocytophilum*, and *B. microti* in individual ticks, underscoring the polymicrobial burden that can be transmitted in a single bite [53,54]. Active tick surveillance in New Jersey documented POWV alongside *B. burgdorferi* and *B. microti* in questing nymphs from the same county in the same season [51]. Tick pathogen surveys from Appalachian Virginia and central Appalachia detected POWV in *I. scapularis* ticks from counties with no prior human case reports, confirming enzootic circulation beyond the recognized endemic boundary [56,57].

Beyond the northeastern corridor, POWV has been isolated from wildlife and ticks across a far broader range—the 1952 *Dermacentor andersoni* isolation in Colorado, a 1987 isolation from a California spotted skunk (*Spilogale putorius*), and serologic evidence in Alaskan and Russian mammals—demonstrating enzootic circulation well beyond the states where human cases concentrate [7,27,58,59]. Established human-pathogenic lineage II (DTV) has been isolated from *I. scapularis* in upstate New York and northern Wisconsin, where tick infection rates are stable over time, and tick-based prevalence surveys aggregated in GIDEON reveal wide geographic variation—from 0% in several Virginia and Long Island collections to 1.3–1.56% in northern Wisconsin and Connecticut, 3.4% in the Hudson Valley, 7.4–10.5% in Maine and on Cape Cod, and up to 16.67% in some Wisconsin pools—underscoring that focal transmission hotspots are embedded within a broader low-prevalence landscape [43,60,61,62,63,64,65,66].

The ecological determinants of POWV transmission intensity are not uniformly distributed even within endemic states. Phylogeographic analyses of DTV sequences reveal highly focal geographic clustering, with viral lineages differing substantially between sites separated by only a few kilometers [13]. Field studies in Maine identified that sites with invasive Japanese barberry (*Berberis thunbergii*) understory maintained significantly higher DTV infection rates in *I. scapularis* than sites with native understory vegetation, attributable to the microclimate stability provided by dense barberry growth—an ecologically important finding linking invasive plant species management to tick-borne disease risk reduction [33]. Wildlife habitat quality, forest fragmentation, and the diversity of mammalian reservoir species (the ‘dilution effect’ hypothesis, whereby high host diversity reduces per-capita tick infection rates) are additional ecological variables that modulate POWV transmission intensity at the local scale [67,68]. These ecological drivers are irreducibly One Health phenomena: they require veterinary, ecological, botanical, and public health disciplines working in concert to characterize, monitor, and address.

Migratory birds represent a final ecological dimension of POWV dispersal that is frequently overlooked in human-focused epidemiological reviews. *I. scapularis* ticks are transported long distances by migratory songbirds, shorebirds, and waterfowl, with infested birds documented carrying ticks across state and national boundaries during spring and fall migration [13,69]. Phylodynamic analyses suggest that south-to-north POWV dispersal through the northeastern United States—documented at approximately 3 km per year on average—is consistent with tick dispersal via migratory birds as a mechanism, though local tick population dynamics also contribute [13]. This avian dispersal pathway means that POWV range expansion cannot be fully understood or modeled without integrating ornithological surveillance data and migratory bird movement ecology—yet another dimension of the One Health web in which POWV is embedded.

Field studies have documented long-distance transport of *I. scapularis* by migratory birds, providing direct evidence that avian movement can introduce the vector to new geographic areas [69].

## 3. Methods

### 3.1. Study Design

This scoping review was conducted in accordance with the Preferred Reporting Items for Systematic Reviews and Meta-Analyses extension for Scoping Reviews (PRISMA-ScR) [70] and the methodological framework of Arksey and O’Malley as refined by Levac et al. [70,71,72]. Scoping review methodology was selected to map the breadth of evidence across the full One Health spectrum of POWV—from ecological and zoonotic drivers through clinical presentation, geographic distribution, demographic risk factors, and coinfection dynamics—without the restrictive eligibility criteria of systematic reviews [73]. This broad scope is essential given the One Health premise that POWV human disease cannot be understood in isolation from the tick vectors, wildlife reservoirs, land use systems, and co-circulating pathogens that collectively generate and distribute human exposure risk. This review is distinguished by its methodological design as an AI-integrative partnership: the primary author contributed six years of curated data collection conducted through official public health sources, local library archival resources, and state-level surveillance systems; the GIDEON (Global Infectious Diseases and Epidemiology Online Network; https://www.gideononline.com/, accessed on 6 June 2026) Infectious Diseases Database provided a secondary source of infectious disease data; while Claude (Anthropic), an artificial intelligence language model, served as an analytical and synthesis AI partner using author- and GIDEON-sourced information and data.

This three-way collaboration—human investigators, clinical AI, and specialized infectious disease database—represents a novel and transparent approach to scoping review methodology. GIDEON was selected based on its recognized data availability, levels of validity, and breadth of coverage. It is a continuously updated, subscription-based infectious disease database covering over 340 diseases across more than 230 countries, including longitudinal outbreak records, epidemiological graphs, country-level case and death data, and diagnostic tools; it was used in this review to provide verified POWV case and death records, comparative tick-borne flavivirus epidemiology, and real-time outbreak alert data not accessible through AI training corpora or standard literature databases [43,74]. Critically, the AI component of this partnership depended entirely on the human investigators to introduce data, documents, and sources that fall outside the AI’s training corpus or temporal knowledge boundary, including state-specific tick-borne disease dashboards, GIDEON-sourced epidemiological records, archival public health reports, and case-level surveillance records from official state health departments. Note that AI language models cannot independently access subscription databases, state health department portals, library archives, or the approximately one-third of this review’s sources that required human curation to obtain [43,74,75,76,77]. This partnership model is described explicitly so that readers and peer reviewers may appropriately evaluate both the scope and the limitations of this methodology, and so that it may serve as a reproducible model for AI-integrative One Health evidence synthesis.

### 3.2. Search Strategy

PubMed/MEDLINE was searched using combinations of “Powassan virus”, “Powassan encephalitis”, “POWV”, “deer tick virus”, “tick-borne flavivirus”, and “arboviral encephalitis United States”. Searches were supplemented with U.S. Centers for Disease Control and Prevention (CDC) surveillance dashboards (ArboNET), state health department communicable disease bulletins from all states with documented POWV activity, peer-reviewed case reports and series, and vetted public health news sources including CIDRAP. All sources through early 2026 were eligible. Importantly, the foundation of the search infrastructure was built by the primary author over a five-year period of systematic data collection, encompassing official public health sources at the federal, state, and local levels, archival records obtained through library-based retrieval, and the GIDEON Infectious Diseases Database—a specialized clinical and epidemiological reference system for infectious diseases globally. State-level tickborne disease surveillance dashboards for forty-eight U.S. states were individually reviewed and catalogued by the authors; these data were then introduced to the AI partner for integration and synthesis, as the AI system does not have independent access to state health department portals, subscription databases, or archival library materials.

### 3.3. Inclusion and Exclusion Criteria

Selection (inclusion) criteria. Sources were selected for inclusion if they reported on one or more of the following: (1) human POWV infection or disease in the United States, including clinical symptoms and outcomes; (2) POWV epidemiology in U.S. human populations; (3) tick vector biology, ecology, or infection prevalence relevant to POWV transmission in the United States; (4) wildlife reservoir host ecology, seroprevalence, or sentinel surveillance for POWV; (5) land use, climate, or environmental drivers of POWV or *I. scapularis* range; (6) co-infection of POWV with other tick-borne pathogens in ticks, wildlife, or humans; (7) One Health frameworks applied to tick-borne flavivirus systems; and (8) comparative ecological and symptom profiles of tick-borne diseases sharing the POWV transmission system.

Rejection (exclusion) criteria. Sources were rejected if they: (1) addressed exclusively animal or laboratory models with no translational relevance to human disease or ecology in the United States; (2) focused solely on non-U.S. geographic contexts without comparative relevance; or (3) addressed POWV molecular genetics only, without ecological or clinical content.

**Figure 3:**
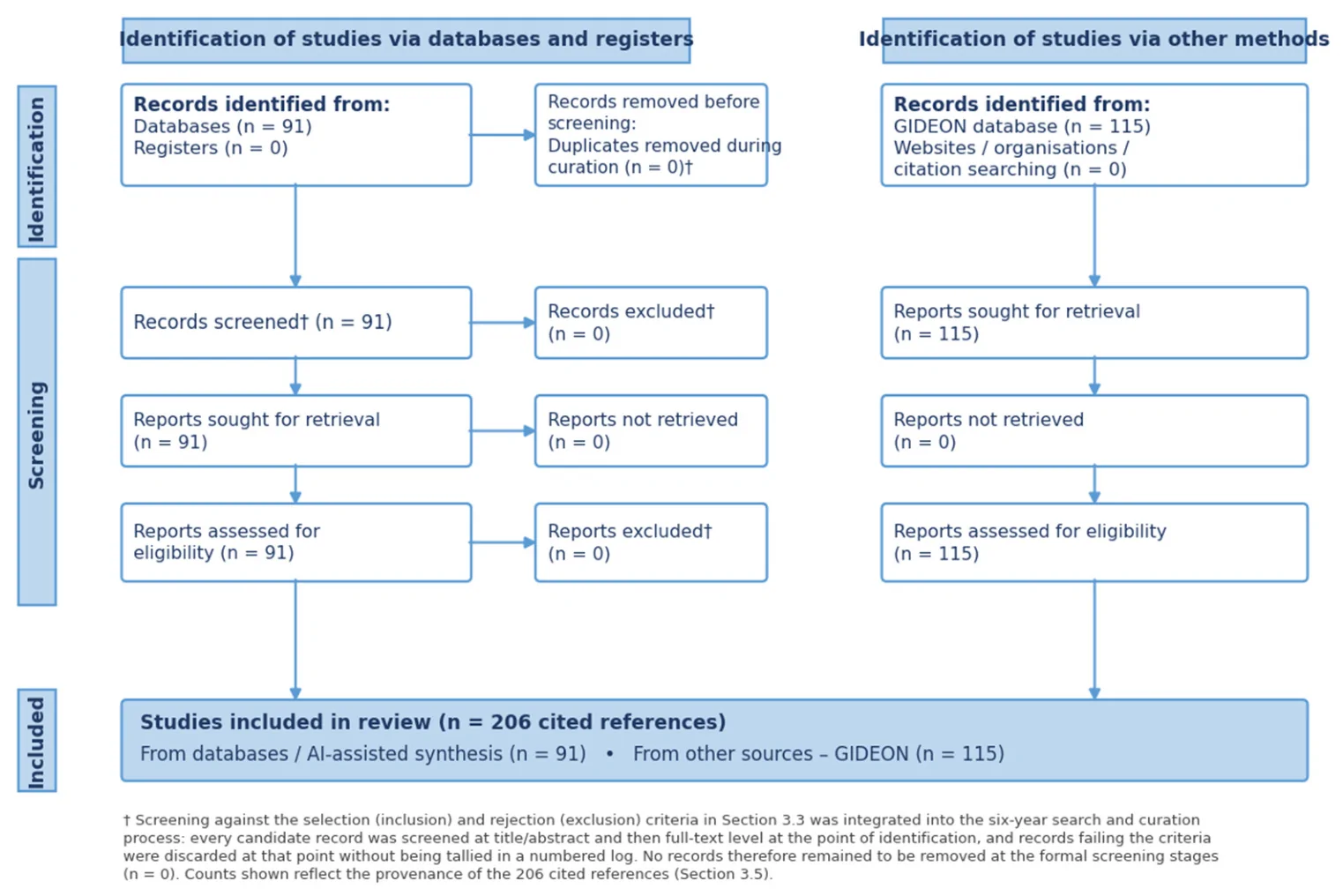
PRISMA 2020 flow diagram for the scoping review, using the PRISMA 2020 template (Table S1) for new reviews that included searches of databases and other sources. Studies identified via databases and registers (**left**) comprise records from PubMed/MEDLINE together with CDC ArboNET, MMWR, state health-department, and public-health news sources integrated through AI-assisted synthesis (*n* = 91); studies identified via other methods (**right**) comprise records from the GIDEON Infectious Diseases Database and author-curated archival and state surveillance records (*n* = 115). Records at every stage were screened against the selection and rejection criteria specified in Section 3.3. Because screening was integrated into the six-year search and curation process, records failing those criteria were discarded at the point of identification and were not tallied in a numbered screening log; no records therefore remained to be removed at the formal screening stages, which are accordingly shown as *n* = 0. The counts shown reflect the provenance of the 206 cited references, as reported in Section 3.5.

Screening process. All candidate records—whether identified through the database searches, AI-assisted synthesis, or GIDEON and archival curation—were screened by the human investigators against these criteria, first at the title/abstract level and then in full text, before being admitted to the source library. Because screening was integrated into the six-year search and curation process rather than performed as a separate pass over a fixed search export, records failing the criteria were discarded at the point of identification and were not tallied in a numbered screening log. Every record entering the formal source library therefore already satisfied the selection criteria, which is why no records are shown as removed at the formal screening stages of the PRISMA flow diagram (Figure 3).

### 3.4. Data Extraction and Synthesis

Data were extracted for each source and organized across the following dimensions: (1) study type and design; (2) geographic scope; (3) patient demographics and ecological exposure pathways; (4) symptom data (prodromal, neurological, laboratory, and imaging findings); (5) clinical outcomes including long-term sequelae; (6) tick vector biology, seasonal activity, and POWV infection rates; (7) wildlife reservoir host species, seroprevalence, and sentinel surveillance data; (8) co-circulating tick-borne pathogens and coinfection evidence; (9) land use, climate, and environmental drivers of POWV risk; (10) One Health policy and surveillance implications; and (11) comparisons with other tick-borne diseases sharing the POWV ecological system. Due to the scoping review design, formal meta-analysis was not performed. Results are presented as a narrative synthesis integrating clinical, ecological, and One Health evidence streams.

### 3.5. Data Provenance and Source Integration

This scoping review synthesized evidence from two complementary streams (Figure 3): the GIDEON Infectious Diseases Database, which supplied curated case records, epidemiological aggregates, and a structured bibliography of the POWV literature, and additional sources identified and integrated through AI-assisted synthesis with Claude (Anthropic), including primary literature, CDC ArboNET and MMWR surveillance reports, and state public health records. Of the 206 references cited in this review, 115 (56%) were sourced from or corroborated by the GIDEON database, while the remaining 91 were independently identified, retrieved, and integrated through the human–AI synthesis process rather than from GIDEON. This distribution illustrates a central methodological finding of this review: that neither a specialized database nor an AI synthesis partner alone produced a complete evidence base, and that the most thorough and accurate picture of POWV emerged only when GIDEON’s curated epidemiological data were integrated with the broader literature and surveillance record through Claude (Anthropic) under human direction [43,74].

**Figure 4:**
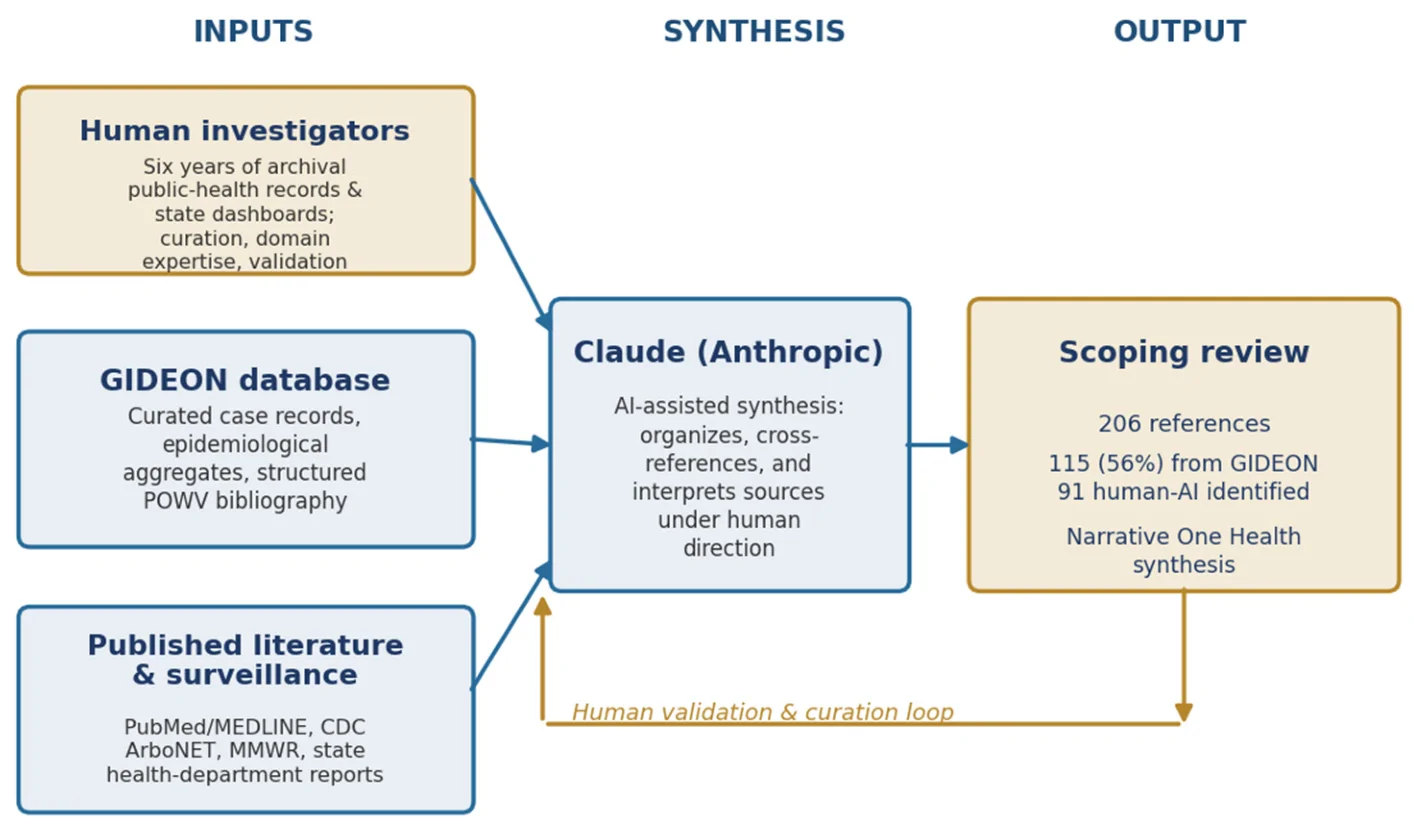
Human–AI–GIDEON collaboration workflow for this scoping review. The human investigators defined the questions, retrieved archival and gray-literature, archival data sources (assembled over approximately six years), and the GIDEON-curated bibliography, and validated all outputs; Claude (Anthropic) organized, cross-referenced, and synthesized these materials and surfaced additional literature for human review. Of the 206 cited references, 115 (56%) were derived from GIDEON and 91 were identified through the human–AI process. Interpretation and final wording remained under human control.

To make the division of labor explicit: the human investigators defined the research questions and search strategy, located and retrieved the archival and gray-literature sources—including state surveillance dashboards and public-health records assembled over approximately six years—supplied the GIDEON-curated case records and bibliography, and verified every extracted figure and citation against its primary source. Claude (Anthropic) was used to organize, cross-reference, and synthesize these human- and GIDEON-supplied materials into structured narrative summaries and to surface additional peer-reviewed literature for human review; it did not independently access subscription databases, state archives, or the GIDEON platform. All AI-generated text and data extractions were reviewed, corrected, and approved by the human authors before inclusion, and the interpretation, conclusions, and final wording remained under human control. This human–AI–GIDEON workflow is summarized in Figure 4.

## 4. Results: Clinical Spectrum of Powassan Virus Infection

### 4.1. Asymptomatic Infection

The most common outcome following POWV exposure is no discernible illness. Seroprevalence studies in endemic states document 0.7–6.1% antibody prevalence in general populations, vastly exceeding the recognized clinical case counts [78]. This gap reflects the One Health reality that the vast majority of human exposure events occur subclinically, likely representing low-level or abortive infections that do not breach the CNS’s barriers [23]. Host immune competence, viral inoculum dose, POWV lineage, and the developmental stage of the biting tick may all modulate the probability of clinical progression [9].

### 4.2. Incubation Period

Symptomatic POWV illness follows an incubation period of 1–5 weeks (range: 7–34 days reported in published cases) [9,79]. This extended and variable window frequently obscures the temporal and geographic link between tick exposure and symptom onset, complicating diagnosis. *I. scapularis* nymphs—responsible for a significant proportion of human bites and only 1–2 mm in size—are routinely overlooked during tick checks, contributing to the frequency with which patients present without recollection of a bite [29].

### 4.3. Non-Neuroinvasive Febrile Illness

Approximately 10% of reported POWV cases present as non-neuroinvasive febrile illness, constituting a substantially underdiagnosed category [9]. Reported non-neuroinvasive cases are laboratory-confirmed by the same criteria applied to neuroinvasive disease under the national arboviral surveillance case definition: detection of POWV-specific IgM antibody in serum with confirmatory plaque reduction neutralization testing demonstrating POWV-specific neutralizing antibodies—or, less commonly, detection of viral RNA by RT-PCR during the brief viremic window—in a patient with a clinically compatible febrile illness and no evidence of central nervous system involvement; confirmed cases are reported to the CDC through ArboNET (see Section 4.6.2) [9,80,81]. Symptoms include fever, headache, myalgia, fatigue, sore throat, and weakness—a profile indistinguishable from influenza-like illness or nonspecific viral syndrome [9,16]. A maculopapular rash has been described in some cases but is neither consistent nor pathognomonic [82,83]. Because clinicians rarely order POWV-specific testing for undifferentiated febrile illness, non-neuroinvasive POWV disease is almost certainly far more common than the surveillance data indicate—a systematic underdetection problem that obscures the true population-level burden of this infection [49].

### 4.4. Aseptic Meningitis

POWV meningitis presents with severe headache, cervical rigidity, photophobia, fever, and malaise, and typically requires hospitalization [84]. Focal neurological deficits are characteristically absent in pure meningitis, though the clinical boundary between meningitis and meningoencephalitis is often difficult to delineate [85]. CSF in POWV meningitis reveals lymphocytic pleocytosis (with possible early neutrophil predominance), normal or mildly elevated protein, and normal glucose [79]. Diffuse EEG slowing may be present even in patients with predominantly meningitic presentations [86]. In the reported cases underlying this profile, POWV meningitis was differentiated from other causes of aseptic meningitis by combined exclusionary and virus-specific testing: negative CSF Gram stain and bacterial cultures excluded bacterial meningitis; molecular testing excluded the more common viral etiologies, notably enteroviruses and herpesviruses; and POWV etiology was established by the detection of POWV-specific IgM in serum or cerebrospinal fluid with confirmatory plaque reduction neutralization testing (see Section 4.6.2) [79,80,81,84]. Epidemiologic context—known or probable tick exposure in an endemic region during the season of tick activity—was typically the feature that prompted clinicians to request POWV-specific testing for an otherwise undifferentiated aseptic meningitis [84].

### 4.5. Neuroinvasive Disease: Encephalitis and Meningoencephalitis

Encephalitis and meningoencephalitis constitute approximately 90% of recognized POWV cases [9,86]. The encephalitic phase typically follows a prodrome of fever, headache, vomiting, myalgia, and fatigue by days to weeks, and can deteriorate precipitously [1]. Key neurological manifestations include:

POWV is classified within the tick-borne encephalitis serocomplex in standard infectious disease references, and the clinical syndrome of POWV neuroinvasive disease closely parallels tick-borne encephalitis virus disease recognized globally, providing comparative clinical context for physicians familiar with international travel medicine [87].

– Altered mental status: Confusion, disorientation, delirium, behavioral abnormalities, and coma in severe cases [85].
– Seizures: Generalized tonic-clonic and focal seizures; subclinical EEG-only seizures may also occur [88].
– Aphasia and dysarthria: Expressive aphasia is a particularly characteristic feature reflecting dominant hemisphere language area involvement [80].
– Paresis and paralysis: Focal limb weakness, hemiparesis, paraparesis, and spastic quadriplegia; documented in 44.1% of adult and 42.6% of pediatric published cases [14].
– Cranial nerve palsies: Ophthalmoplegia, facial weakness, diplopia; reported as both acute and long-term findings [89].
– Ataxia and tremors: Reflecting cerebellar and subcortical involvement [90].
– Respiratory failure: Acute hypoxemic respiratory failure requiring mechanical ventilation in the most severe cases, correlating strongly with mortality [85].
– Rhombencephalitis (brainstem encephalitis): A recognizable brainstem-predominant form of POWV neuroinvasive disease, presenting with cranial neuropathies, ophthalmoplegia, dysphagia, ataxia, and early respiratory compromise. It constitutes a distinct clinical entity that may occur with or without diffuse cerebral involvement; its brainstem-centered MRI signature can mimic other rhombencephalitides and complicate diagnosis [91].
– A published case report from New York illustrates this trajectory: a patient with expressive aphasia and progressive obtundation required mechanical ventilation and was left with substantial neurological deficits despite ICU-level supportive care [80]. Atypical neuroimaging patterns, including rhombencephalitis with predominant brainstem involvement, have been reported and highlight that POWV can produce unusual CNS distribution patterns that complicate diagnosis [91].

Elevated intracranial pressure, hyponatremia from SIADH, and systemic inflammatory responses represent serious comorbid complications of severe POWV encephalitis [55,85].

### 4.6. Diagnostic Findings

#### 4.6.1. Cerebrospinal Fluid

CSF in POWV encephalitis reveals lymphocytic pleocytosis (early neutrophil predominance possible), normal-to-mildly elevated protein, and normal glucose [79]. Standard multiplex CNS PCR panels (e.g., BioFire FilmArray ME Panel) do not include POWV; a normal panel does not exclude it [86]. This CSF profile also helps distinguish POWV from other *Ixodes*-transmitted diseases: Lyme neuroborreliosis also produces a lymphocytic pleocytosis, but typically with higher protein and intrathecal production of Borrelia-specific antibody, whereas anaplasmosis rarely involves the central nervous system and usually leaves the CSF normal or only minimally abnormal, so a pronounced lymphocytic pleocytosis with normal glucose in a patient with tick exposure favors a viral encephalitis such as POWV over anaplasmosis [79,92].

#### 4.6.2. Serology

POWV-specific IgM ELISA or IFA testing of serum and/or CSF constitutes the diagnostic standard [81]. Detection of POWV IgM in CSF is particularly confirmatory of CNS infection [80]. Confirmatory plaque reduction neutralization testing (PRNT) requires weeks to result [84]. POWV serological testing is available commercially, at some state public health laboratories, and at CDC; positive IgM results should be confirmed by PRNT at a state public health laboratory or CDC [93,94].

A comprehensive review of tick-borne disease diagnostics confirms that POWV remains absent from commercially available multiplex panels, requiring clinicians to specifically request state-laboratory POWV serology and preventing routine panel-based exclusion of the diagnosis [95]. Cerebrospinal fluid metagenomics using next-generation sequencing has demonstrated added diagnostic value specifically for POWV in cases where conventional serology is indeterminate [96]. POWV serologic diagnosis can be complicated by antibody cross-reactivity among antigenically related flaviviruses—such as West Nile virus and other members of the tick-borne encephalitis serocomplex—which may yield cross-reactive POWV IgM ELISA results that require plaque reduction neutralization testing for resolution in regions where multiple flaviviruses co-circulate [81]. Jamestown Canyon virus, an orthobunyavirus, is not serologically cross-reactive with the flavivirus POWV; rather, because it co-circulates in the same geographic regions and can cause a clinically similar neuroinvasive febrile illness, background Jamestown Canyon virus seropositivity has been described as a source of diagnostic confusion, and the two infections are best distinguished on clinical grounds and virus-specific serology rather than by neutralization testing to resolve serologic cross-reactivity [97].

### 4.7. Long-Term Neurological Sequelae

Approximately 50% of POWV neuroinvasive survivors experience long-term neurological deficits [21]. Reported sequelae include persistent headaches, muscle weakness and wasting, focal paralysis, cognitive impairment, persistent aphasia, ataxia, ophthalmoplegia, and personality changes [90]. Factors independently associated with persistent deficits include: ataxia (r = 0.383, *p* = 0.006), paralysis (r = 0.278, *p* = 0.048), speech disorder (r = 0.319, *p* = 0.022), and cranial nerve involvement (r = 0.322, *p* = 0.017) [14]. The biological basis appears to involve chronic neuroinflammation and persistent viral RNA rather than active replication—a mechanism relevant to future therapeutic development [42]. The One Health dimension of these sequelae extends to societal impact: POWV survivors often require prolonged rehabilitation, caregiver support, and loss of occupational function, imposing substantial burdens on families, healthcare systems, and communities in endemic regions [98].

Whether co-infection with other *Ixodes*-transmitted pathogens capable of neuroinvasion worsens the clinical course or long-term outcome of POWV is not well-studied. Concurrent Lyme neuroborreliosis, and the systemic inflammation of severe babesiosis or anaplasmosis, could plausibly compound acute neurological injury, prolong recovery, and worsen residual deficits; conversely, the treatable co-infections may be controlled while POWV is not. Direct evidence remains limited, and the effect of polymicrobial tick-borne infection on neuroinvasive outcomes is a priority for future study [99,100].

Immunocompromised patients face particular risk for severe and atypical POWV disease; a published case series from an immunocompromised host illustrated diagnostic delays attributable to atypical MRI patterns and the absence of traditional clinical risk factor recognition [101]. Patients with autoimmune conditions may face compounded risk: neutralizing auto-antibodies against type I interferons, documented in patients with severe tick-borne flaviviral infections including POWV, suggest that immune profiling of at-risk individuals could inform personalized prevention counseling [102]. Children with POWV encephalitis frequently require prolonged hospitalization, neurorehabilitation, and ongoing educational support, with outcomes affecting developmental trajectories for years after acute illness [103].

## 5. Geographic Distribution: A One Health Ecological Map

As shown in Figure 1, POWV cases are concentrated in the northeastern states and Great Lakes region, reflecting the geographic range of the primary vector, *Ixodes scapularis*. States reporting the highest cumulative case counts include Minnesota, Wisconsin, New York, Massachusetts, and Maine. Viral evidence in ticks and wildlife extends beyond confirmed human case states to include the mid-Atlantic, southern, and western states.

### 5.1. Northeast and Great Lakes Core Endemic Regions

POWV disease in the United States is geographically concentrated in the northeastern states and Great Lakes region (Figure 1), reflecting not simply the distribution of its primary vector *I. scapularis*, but the full ecological system that sustains transmission: high-density white-footed mouse populations, forested and forest-edge habitat, sufficient deer density to support tick reproduction, and the temperature and humidity conditions that support tick survival and questing activity [12,15,31,33]. Geographic variation in human case incidence therefore mirrors variation in this ecological complex rather than merely the distribution of a single species. During 2006–2016, Minnesota (*n* = 32), Wisconsin (*n* = 22), New York (*n* = 16), and Massachusetts (*n* = 16) reported the highest case counts nationally [9]. In New York State (2013–2023), 44 cases were reported; the highest incidence occurred in Columbia and Putnam counties, correlating with elevated *I. scapularis* density and suburban–forest interface land use [9,104]. Hospitalization was required in 91% of New York cases; 11% were fatal [104]. In New England, an eight-patient case series documented presentations including headache, fatigue, diplopia, word-finding difficulty, seizures, and one fatal encephalitis within 18 days of illness onset [82].

The northeastern endemic focus was formally recognized in the early 2000s when a cluster of Powassan encephalitis cases in Maine and Vermont prompted a CDC investigation and a formal MMWR outbreak report, establishing these two states as the initial epicenters of recognized human POWV disease in the United States [105,106,107].

Wisconsin reported 12 cases in 2024, second nationally to Minnesota (14 cases), with cases concentrated in the northern, forested half of the state where *I. scapularis* is most abundant [108,109]. Minnesota has consistently led the nation in case counts; a Minnesota case series documented presentations including intraparenchymal hemorrhage, seizure, altered mental status, and severe headache [110].

Neitzel and colleagues described a 2013 Minnesota case that illustrated both the severity of POWV disease and the challenges of clinical recognition in a community hospital setting [111]. ProMED and other case report alerts from New York State from 2013 through 2019 further documented recurring case recognition in the Northeast as surveillance matured [112,113,114,115,116,117,118,119,120].

### 5.2. Expanding Geographic Range: Mid-Atlantic and Southern States

From a One Health perspective, geographic expansion beyond the traditional endemic core is one of the most significant epidemiological trends in POWV [5]. New Jersey (7 cases, 2008–2017) ranks fifth nationally and was the site of at least one fatal case [121]. Pennsylvania reported five cases in the 2008–2017 period, and a 2025 case report documented POWV encephalitis in western Pennsylvania—a county not previously considered endemic—attributed to expanding *I. scapularis* range driven by climate change [17]. Virginia has recorded at least one confirmed case and has documented enzootic POWV as far south along the eastern seaboard as the Virginia coastline [23].

Systematic arbovirus surveillance across Virginia counties documented widespread POWV-reactive antibodies in wildlife and tick populations in multiple counties, including areas not previously considered at risk [63]. In Appalachian Virginia, Cumbie and colleagues documented the first evidence of POWV in *I. scapularis* from the southern Appalachians, suggesting active southward expansion of the enzootic footprint [122]. In that Virginia survey (2020–2022), plaque-reduction neutralization testing detected POWV-neutralizing antibodies in 49.8% of white-tailed deer (127 of 255) and 1.0% of cattle (5 of 500), as well as in smaller numbers of Virginia opossums (8.1%), northern raccoons (2.7%), red foxes (2.5%), and eastern cottontail rabbits (1.5%), demonstrating that POWV exposure is distributed across a broad range of wildlife and livestock hosts in a state with few recognized human cases [43,63].

North Carolina and Tennessee represent the southernmost documented extensions of confirmed human POWV disease in the published literature [14,23,123]. North Carolina appeared in the 13 states reporting confirmed cases to the CDC in 2019. A systematic review of published POWV cases cited Tennessee as the southernmost confirmed case location. Indiana also appeared among the 2019 reporting states [124]. The Indiana State Department of Health acknowledges that local versus travel-associated transmission cannot always be definitively determined from case reports—a surveillance complexity common to outlier states [125].

Ohio documented POWV disease in a case identified through metagenomic next-generation sequencing, representing one of the state’s earliest recognized human infections and prompting heightened surveillance in the upper Midwest [126]. Illinois subsequently reported a resident diagnosed with POWV in 2025, prompting statewide tick testing [127]. Rhode Island’s first confirmed POWV neuroinvasive disease case was reported in 2018 [128]; additional 2021 reports from ProMED and the Rhode Island Department of Health documented a subsequent case [129,130].

Delaware has been identified by state authorities as a state with emerging POWV risk, with public health communications alerting residents following the identification of local POWV-competent tick populations [131]. National tick-borne disease awareness commentaries have noted that the public recognition of POWV’s expanding range remains critically low outside established endemic states, potentially contributing to underdiagnosis in the mid-Atlantic and southern states [132].

### 5.3. Western States: Viral Evidence Without Human Cases

The most striking evidence of POWV’s continental reach comes from the western United States, where viral isolations document circulation in the absence of reported human cases [58]. Colorado holds historical primacy: the 1952 isolation of a POWV-related virus from a *D. andersoni* tick along the Cache la Poudre River predated the virus’s human characterization and is now recognized as the prototype lineage II (DTV) isolate [7]. Colorado designates POWV disease as a state-reportable condition but has no confirmed human cases, attributed in part to the absence of *I. scapularis* within its state borders [133]. POWV was isolated from a spotted skunk in California in 1987 [59].

Serological surveys of snowshoe hares in Alberta, Canada detected POWV-reactive antibodies, establishing viral presence in western Canada, well outside the primary human endemic zone [48]. Deardorff and colleagues systematically documented POWV in mammals from Alaska and New Mexico as well as in Russia, confirming a broad intercontinental distribution that exceeds recognized human disease boundaries [27].

From a One Health perspective, these western findings indicate that POWV enzootic cycles have historically been maintained well beyond the human disease reporting boundaries. A plausible explanation for this human–animal disconnect lies in vector ecology: the western and subarctic enzootic cycles are maintained by ticks such as *D. andersoni* and nidicolous (nest-dwelling) *Ixodes* species whose hosts are principally rodents, lagomorphs, and mustelids and which seldomly bite humans, whereas the eastern epicenter of human disease coincides with the range of *I. scapularis*—an aggressive generalist tick that readily feeds on people—so the virus can circulate enzootically where no efficient bridge vector delivers it to humans [7,25,26,27,58]. Diagnostic and surveillance bias may compound this gap: where POWV is not perceived as a local threat, clinicians rarely request the specialized send-out testing required to detect it, so sporadic human infections could escape recognition [49,133]. They also underscore the importance of wildlife and tick surveillance as leading indicators of human disease potential—a One Health surveillance principle that is currently underutilized for POWV across much of the country [49].

### 5.4. Seasonality and Climate-Driven Trends

POWV cases peak in late spring and early summer (April–June), reflecting *I. scapularis* nymph and adult activity [134]. However, cases have been documented in winter months, and the conventional assumption that tick-borne disease is a warm-season phenomenon represents a diagnostic hazard [86]. *I. scapularis* range has expanded at approximately 46 km per decade, driven by climate warming [31]. Phylodynamic analyses confirm that DTV population expansion is concordant with *I. scapularis* ecological expansion [31]. Climate projections anticipate continued northward displacement of tick habitat, implicating climate change as a structural driver of POWV epidemiological growth—a canonical One Health issue demanding cross-sectoral policy responses [17].

A systematic review of climate change and tick-borne disease in the United States confirmed that warming temperatures, reduced frost days, and changing precipitation patterns are primary drivers of *I. scapularis* range expansion, with POWV identified as among the tick-borne pathogens most likely to expand in tandem [32]. This finding can be extrapolated directly to the geographic distribution of POWV documented in this review (Figure 1; Section 5.1, Section 5.2 and Section 5.3): the case concentrations in the Upper Midwest and Northeast—Minnesota, Wisconsin, and northern New England, including Maine’s nation-leading per-capita incidence—lie along the northern latitudes where warming-driven *I. scapularis* expansion has been most pronounced, and the recent accession of first-ever cases in every New England state, together with the record Maine (2023) and national (2024) case counts, is temporally consistent with that climate signal (Section 5.1 and Section 5.7). By the same logic, continued warming would be expected to convert regions that currently show only enzootic or emerging evidence—the mid-Atlantic and northern-latitude areas described in Section 5.2 and Section 5.3—into case-reporting areas as vector density and human–tick contact increase; the western enzootic foci, maintained by non-*I. scapularis* vectors, are the principal exception to this extrapolation. This anticipatory logic underpins the surveillance expansion argued in Section 5.5 [31,32,135]. Climate-driven changes in reservoir host distributions, particularly deer and white-footed mouse range expansions, further amplify risk by enlarging the geographic scope of communities available to sustain enzootic transmission [135]. The WHO, FAO, OIE, and UNEP jointly recognize tick-borne zoonotic diseases as priority targets for multisectoral One Health action, a framework directly applicable to POWV’s climate-driven range expansion [136].

### 5.5. One Health Implications of Geographic Distribution

The geographic pattern of POWV in the United States is not a fixed entity but an ecological process—one being actively reshaped by climate, land use, wildlife population dynamics, and tick dispersal via migratory birds [13]. A One Health approach to POWV geographic risk requires integrating: (1) entomological surveillance of *I. scapularis* density and infection prevalence; (2) wildlife seroprevalence monitoring as an early-warning sentinel system; (3) environmental modeling of habitat suitability under climate scenarios; and (4) human case surveillance stratified by ecological exposure class. Fourteen states now report POWV cases to the CDC, but viral evidence in ticks and wildlife suggests that the risk boundary is substantially broader [12]. Clinicians in non-traditional-endemic states—particularly the mid-Atlantic, southern, and interior-northern United States—should maintain POWV on their differential diagnosis for unexplained acute encephalitis, especially when patients have traveled to high-endemicity areas [37].

### 5.6. Ecological Determinants of Geographic Heterogeneity in POWV Risk

POWV risk is spatially heterogeneous even within endemic states. Phylogeographic analysis shows that DTV lineages cluster tightly by site, differing over as little as a few kilometers, so transmission is concentrated in discrete hotspots—some no larger than a residential property—embedded in a broader low-risk landscape; because most exposure risk arises from this small fraction of the landscape, targeted ecological intervention at such foci—vegetation management, deer population control, and habitat modification—is expected to reduce human infections in proportion to the concentrated share of transmission risk that these sites carry, averting a large fraction of infections while requiring treatment of only a limited area [13,137].

These hotspots are shaped by interacting ecological variables: forest microclimate (*I. scapularis* nymphs require ≥85% humidity, favoring closed-canopy, leaf-litter-rich sites, and dense invasive Japanese barberry thickets), mast-driven boom–bust cycles in white-footed mouse abundance, and forest fragmentation that raises mouse density by reducing predator diversity (the dilution effect) [33,67,68]. Because two nearby properties can differ sharply in transmission intensity, One Health ecological monitoring of these variables could enable finer risk mapping than human case data alone. Earlier work documented a focal deer tick virus transmission focus in the northcentral United States [138], while clinical criteria distinguishing DTV encephalitis from herpes simplex and Lyme neuroborreliosis have also been described [38,139].

### 5.7. County-Level Surveillance Data and Deaths: ArboNET Evidence Base

The CDC ArboNET surveillance system—the national arboviral surveillance infrastructure maintained jointly by the CDC and state health departments—provides the most authoritative, standardized, and temporally complete dataset on POWV human disease in the United States [140]. ArboNET data are reported by state health departments using standard surveillance case definitions and stratified by neuroinvasive versus non-neuroinvasive presentation, hospitalization status, death, and geographic unit of residence. The CDC Historic Data Dashboard encompasses human POWV data from 2004 through 2025, with 2025 data designated preliminary and current as of 2 June 2026; the Current Year Data dashboard provides preliminary 2026 season data updated every one to two weeks [11]. Although the ArboNET county-level interactive dashboard presents incidence maps and case counts at the county resolution, the system’s own documentation acknowledges that reported cases represent a significant underestimate of true burden, as the passive surveillance architecture depends on clinician suspicion, appropriate diagnostic ordering, and timely reporting [140]. With those limitations stated explicitly, the county-level data available through ArboNET and corresponding state health department dashboards constitute the most granular empirical base currently available for characterizing POWV’s geographic footprint.

#### 5.7.1. National Case Trajectory and the 2023–2024 Surge

Annual U.S. POWV case counts reported to ArboNET demonstrate a staircase pattern of escalation punctuated by year-to-year variability: 1 case in 2004, rising irregularly to a then-record 22 in 2016, 34 in 2017, 43 in 2019, 47 in 2022, and 49 in 2023—the latter representing, at that time, the highest single-year total since POWV became separately reportable in 2004 [11,109,141]. The 2023 MMWR report (Padda et al., 2025) documented 49 cases from 11 states; 47 (96%) were neuroinvasive, 44 (90%) were hospitalized, and 8 (16%) died [142]. The median age of the 2023 cases was 68 years, and 65% were male [142]. The 2023 record was broken the following year: in 2024, final ArboNET data documented 60 cases, all hospitalized with neuroinvasive disease, including nine deaths, from 10 states and 45 counties [109]. Minnesota led in 2024 with 14 cases; Wisconsin reported 12—a state record; Massachusetts, Maine, and other northern East Coast states accounted for the remainder [9,13,108,109].

The 2024 final ArboNET data confirmed that 45 individual counties across 10 states reported at least one human POWV disease case [109]. This county-count is itself a meaningful surveillance metric: it reflects the geographic spread of active POWV transmission beyond the handful of high-incidence counties that dominated earlier surveillance periods. In the 2006–2016 period, POWV cases were reported from only nine states (Maine, Massachusetts, Minnesota, New Hampshire, New Jersey, New York, Pennsylvania, Virginia, and Wisconsin), and the average annual incidence nationally was 0.0025 per 100,000 persons [9]. By 2023–2024, that footprint had grown to include Connecticut, Rhode Island, Maryland, North Carolina, Indiana, Ohio, North Dakota, and sporadic other states, reflecting both ecological expansion and improved surveillance capacity [11,124,141,142].

#### 5.7.2. State Incidence Leaders: Per-Capita Burden at the State and County Level

When POWV burden is expressed as incidence per 100,000 population—the metric used in MMWR annual arboviral summaries and the CDC county-level incidence map (2006–2015)—the picture differs substantially from the raw case counts [11,142]. Maine, New Hampshire, and Vermont emerge as the highest-incidence states nationally, despite their relatively modest absolute case numbers, because their small populations amplify per-capita rates. In 2023, Maine reported a neuroinvasive POWV incidence of 0.50 per 100,000 population, New Hampshire 0.29, and Vermont 0.15—the three highest state-level rates in the United States that year [142]. These figures substantially exceed the national Powassan virus disease incidence and underscore that for a clinician practicing in rural northern New England, POWV is not a theoretical concern but an active, present risk.

The CDC’s county-level average annual incidence map for 2006–2015—which shades counties into three incidence tiers (below 0.20, 0.20–0.49, and at or above 0.50 per 100,000)—concentrates the highest-shaded counties primarily in northern Minnesota, northern Wisconsin, and scattered New England counties, with lower-incidence shading extending through the Hudson Valley of New York and coastal New England [14]. That map, sourced from ArboNET data and available from the CDC Stacks repository and the historic data dashboard [11], represents the most granular public-domain county-resolution incidence visualization for POWV in the United States. The CDC recommends the interactive ArboNET Historic Data Dashboard (https://www.cdc.gov/powassan/data-maps/historic-data.html, accessed on 26 June 2026) as the primary reference for current county-level case data as it allows filtering by year, state, and case type [11].

From 2006 through 2015, state-level ArboNET data show Minnesota (*n* = 20), Wisconsin (*n* = 16), New York (*n* = 16), Massachusetts (*n* = 8), New Jersey (*n* = 3), New Hampshire (*n* = 1), Pennsylvania (*n* = 1), Virginia (*n* = 1), and Maine (*n* = 2) as the reporting states [9,11]. By the 2008–2017 period, case counts had grown to Minnesota (32), Wisconsin (22), New York (16), Massachusetts (16), New Jersey (7), Maine (6), Pennsylvania (5), New Hampshire (3), Rhode Island (3), Connecticut (1), North Carolina (1), North Dakota (1), and Virginia (1) [11,124]. These sequential tallies, drawn from ArboNET and compiled by state epidemiologists, document both the persistence of the same core endemic states and the gradual accretion of new reporting states over time.

#### 5.7.3. State County-Level Profiles: Highest-Burden Localities

Table 1 summarizes the distinctive epidemiological characteristics of the principal POWV-reporting states, allowing regional differences in burden, geography, and notable events to be compared directly [9,58,89,104,108,133,143,144,145].

#### 5.7.4. Death Data: Surveillance of Fatal POWV Cases by State

Death data are the most completely reported stratum of POWV surveillance, because fatal cases are almost always hospitalized and thoroughly investigated. Attribution of these deaths to POWV is laboratory-grounded rather than inferential: surveillance cases meet standardized clinical and laboratory criteria, which may include POWV-specific IgM with neutralizing antibody confirmation, RT-PCR, immunohistochemistry, or molecular testing of tissue, and fatality status is reported through ArboNET by state health departments using standardized surveillance fields [9,94,142,146]. From 2004 through 2022, 36 out of 288 reported cases (12.5%) were fatal and 264 (91.7%) were hospitalized; in 2023, 8 out of 49 (16%) died at a median age of 71 years [11,142]. Fatality is consistently age-stratified—the 2006–2016 analysis found all deaths in patients over 50—and a tertiary-center series (Mayo Clinic, 2013–2022) reported 90-day mortality of 18.8% with residual deficits in 72.7% of survivors [9,88]. Even so, POWV accounts for only about 0.3% of encephalitis in North America, a reminder that recognized burden remains small even as it grows [43].

The changing threat profile reflects the continued northward and range-wide expansion of *Ixodes scapularis*—whose recorded U.S. distribution more than doubled between 1996 and 2015—together with rising incidence of the anaplasmosis, babesiosis, and Powassan virus infections it transmits. Powassan virus, including the deer tick virus subtype, has been identified as a growing concern, and without sustained advances in surveillance, diagnostics, and vector suppression this trend is likely to continue. State-level records add geographic granularity: Maine has documented four deaths since 2015, Minnesota fatal cases in 2011, 2021 (two), and 2022, Wisconsin and New Jersey at least one each, and Maryland’s first-ever case in 2023 was a fatal, travel-associated infection [16,35,104,110,147]. ProMED alerts, state press releases, and local news constitute an underutilized secondary surveillance stream that captures such fatalities in near real-time, ahead of ArboNET finalization. This complementary approach may be particularly valuable for rare arboviral infections such as Powassan virus disease, for which small case numbers magnify the impact of reporting delays, but similar limitations have been documented for other ArboNET-tracked diseases, including West Nile, La Crosse, and Oropouche virus disease, where state and local data may precede national reporting and provisional surveillance data can lag weeks behind disease onset [148,149,150,151,152,153,154,155,156,157,158,159,160,161,162,163,164,165,166,167,168,169,170,171].

#### 5.7.5. CDC Maps as Primary Reference: Guidance for Clinicians and Researchers

Three CDC-published or peer-reviewed maps constitute the authoritative visual reference for POWV geographic distribution, as noted in the Figure 1 legend in this review [11,12,13,14]. The CDC Historic Data Dashboard (https://www.cdc.gov/powassan/data-maps/historic-data.html, accessed on 6 June 2026) provides an interactive county-level POWV case and incidence visualization for 2004–2025; 2025 data are preliminary and the data are current as of 2 June 2026 [11]. This dashboard allows users to filter by year, state, case type (neuroinvasive vs. non-neuroinvasive), and death, and renders county-shaded maps and downloadable data tables. The CDC’s 2006–2015 static county incidence map—available through CDC Stacks (stacks.cdc.gov) and the archived statistics page—shades counties into three incidence tiers (below 0.20, 0.20–0.49, and at or above 0.50 per 100,000) and concentrates the highest-shaded counties in northern Minnesota, northern Wisconsin, and New England [11]. A complementary peer-reviewed map by Hassett and Thangamani (2021) in *Microorganisms* showed the 14 states reporting POWV cases to ArboNET alongside a trend graph [12]. Vogels et al. (2023) in PNAS presented a phylogeographic map of DTV lineages across the northeastern United States, providing molecular-epidemiological geographic context [13]. Kakoullis et al. (2023) in Tropical Medicine and Infectious Disease mapped POWV case distribution overlaid with *I. scapularis* range [14]. Together, these maps—interactive federal surveillance visualizations, static peer-reviewed figures, and phylogeographic reconstructions—compose a multi-resolution geographic evidence base that clinicians, public health practitioners, and researchers should consult in tandem when characterizing current POWV risk by county.

#### 5.7.6. Surveillance Limitations and the Undercount Problem

The county-level data reviewed here are subject to limitations that must be stated to avoid misinterpretation. ArboNET is a passive surveillance system dependent on (1) clinicians considering POWV in the differential diagnosis; (2) appropriate diagnostic test ordering; (3) testing through commercial, state public health, or federal laboratories, with confirmatory testing often performed by public health laboratories; and (4) case reporting by health departments [11,94,140,146]. Each step represents a filter that preferentially removes mild and asymptomatic cases from the record. Seroprevalence studies in Maine documented 1.1% POWV antibody prevalence in residents bitten by *Ixodes ticks* [20], and studies in endemic areas of the Northeast have documented a 0.7–6.1% population seroprevalence [19,20], vastly exceeding any reasonable interpretation of the official case counts. This means that the 45 counties that reported POWV cases to ArboNET in 2024 almost certainly underrepresent the counties with active transmission; they represent the counties where transmission occurred, was severe enough to prompt hospitalization and testing, and where testing and reporting followed through the full chain.

Geographic underreporting is compounded by testing availability disparities. In states without POWV-experienced academic medical centers or state laboratories with validated POWV immunoassays, clinicians may not order or have access to POWV-specific testing even when the clinical picture is compatible. The multiplex FilmArray CNS panel, now widely used in emergency encephalitis workups, does not include POWV, meaning that a negative panel does not exclude the diagnosis [86]. Clinicians in non-traditional-endemic states who lack familiarity with POWV may exhaust the standard encephalitis workup without ever ordering POWV serology. The result is that the county-level map—both the ArboNET interactive version and the static CDC/ArboNET publications—should be read as a map of where POWV was detected and reported, not as a definitive delineation of where POWV is absent. As *I. scapularis* continues its documented range expansion and as diagnostic awareness improves, the filled counties on that map are expected to multiply.

These limitations underscore the need to highlight population-based serosurveillance as the most direct means of establishing the true endemicity of POWV. Because passive, case-based ArboNET reporting captures only the small fraction of infections severe enough to prompt hospitalization and testing, it systematically underestimates where the virus is actually endemic. Structured serosurveys of human populations bypass this filter by measuring cumulative exposure directly: a seroprevalence of 0.7–6.1% in endemic areas of the Northeast, 1.1% among tick-bitten Maine residents, and a multi-state blood-donor serosurvey that detected POWV-specific neutralizing antibodies in 0.9% of donors—with county-level estimates as high as 11.5%—together reveal an endemic footprint far larger than the reported case counts imply [17,19,20,172]. The Sussex County, New Jersey investigation is illustrative: following a cluster of neuroinvasive cases, a community seroprevalence study estimated that roughly 23% of infections progress to neuroinvasive disease, so the confirmed cases could be back-calculated to a much larger pool of undetected community infection [145]. Systematic, geographically stratified human serosurveillance—integrated with the entomological and wildlife-sentinel surveillance emphasized throughout this review—would provide the population-level denominator needed to define POWV endemicity, target prevention, and distinguish areas of true absence from areas of merely absent surveillance.

## 6. Age-Related Patterns in Powassan Virus Symptoms and Outcomes

### 6.1. Age Distribution

POWV neuroinvasive disease affects all age groups with a bimodal pattern of elevated risk: older adults and young children [9]. In the 2017 national surveillance, 56% of cases were in patients aged 60 or older and 15% in those under 18 years [173]. In New York State (2013–2023), patients over 50 accounted for 65.9% of cases; children under 10 accounted for 20.5%, including two infant cases (aged 23 days and 1 month) [104]. The 1999–2005 national series recorded a median age of 69 years [8]. These demographic patterns reflect a combination of behavioral exposure differences, age-related biological susceptibility, and immunological factors.

### 6.2. Older Adults (Age ≥ 60)

Older adults bear the highest burden of severe disease and death. Mortality is significantly associated with older age (r = 0.264, *p* = 0.029) in published case series [14]. Experimental murine models demonstrate that POWV lethality increases more than tenfold with age, driven by age-dependent divergence in neuroinflammatory cytokine profiles and glial cell activation [41]. Clinically, older POWV patients frequently exhibit rapidly progressive encephalopathy and are more likely to have comorbidities—including malignancy, immunosuppression, diabetes, and cardiovascular disease—that compound neuroinflammatory injury [174]. From a One Health lens, the aging of the U.S. population in endemic rural and suburban areas will increase the proportion of high-risk individuals in tick-exposed environments, amplifying future case severity [16].

A central mechanism underlying this age gradient is immunosenescence—the progressive decline and dysregulation of innate and adaptive immunity with age. Reduced naïve T-cell output, impaired type I interferon and antiviral responses, and chronic low-grade inflammation (“inflammaging”) together blunt the early control of viral replication while amplifying the neuroinflammatory injury that drives POWV lethality. Immunosenescence thus helps explain the rapidly progressive encephalopathy, higher case fatality, and poorer functional recovery observed in older adults, and it compounds the effects of the comorbidities common in this group [41,174].

### 6.3. Middle-Aged Adults (Age 18–59)

Middle-aged adults represent the second largest case group and include working-age individuals engaged in outdoor occupational and recreational activities—a behavioral exposure factor directly relevant to One Health [134]. Case reports document severe encephalitis in this group, including a 55-year-old presenting with acute confusion and symmetric basal ganglia MRI lesions, and a 62-year-old with rapidly progressive expressive aphasia requiring intubation [80,175]. Immunocompromising conditions—including chemotherapy, transplant immunosuppression, and autoimmune disease treatments—substantially increase the risk in this age group, and POWV has been transmitted via blood transfusion in at least one documented U.S. case involving a kidney transplant recipient [176,177].

### 6.4. Pediatric Cases (Age < 18)

Pediatric POWV deserves specific characterization. A systematic review identified 14 pediatric published cases (16.7% of total published cases) [14]. Paralysis was documented in 42.6% and cognitive deficits in 25% of pediatric cases [14]. Pediatric mortality (7.1%) was substantially lower than adult mortality (19.1%) [14]. Spinal cord involvement appears notably in pediatric case reports: a nine-year-old with bilateral basal ganglia MRI hyperintensities and cervical/mid-dorsal spinal cord T2 hyperintensities required ICU admission and hypertonic saline for elevated intracranial pressure [14,178,179]. Two confirmed infant cases—including a 23-day-old—in New York State highlight that even newborns are susceptible, though the mechanisms of neonatal acquisition remain poorly characterized [104].

A 2025 case series by Greenlaw and colleagues documented pediatric POWV encephalitis in New England, describing variable presentations including seizures, movement disorders, and prolonged ICU courses in children as young as school age, with long-term neurodevelopmental follow-up identified as essential for all pediatric survivors [103]. An infant on Martha’s Vineyard, Massachusetts, contracted POWV following a tick bite, highlighting that even very young infants in endemic communities face risk and prompting expanded public awareness targeting parents of young children [180]. A systematic review of tick-borne infections in children in North America identified POWV as an increasingly important pediatric diagnosis that is likely underrecognized due to its exclusion from standard multiplex panels [181]. Emergency and wilderness medicine guidelines now specifically include POWV in the differential for pediatric patients presenting with acute febrile illness and tick exposure in endemic areas [182].

From a One Health perspective, pediatric exposure often occurs through recreational and family-based outdoor activities—camping, hiking, gardening—that represent contexts for environmental health education interventions in endemic communities [26]. Schools and pediatric primary care providers in endemic areas represent important channels for One Health-informed tick exposure prevention education.

## 7. Sex-Based Patterns in Powassan Virus Disease

### 7.1. Male Predominance

A consistent male predominance characterizes POWV surveillance data. In the 2017 national surveillance, 82% of POWV cases were male—the highest male-to-female ratio among all nationally reported arboviral diseases that year, exceeding West Nile virus (62%), Jamestown Canyon virus (61%), and La Crosse virus (54%) [173]. New York State (2013–2023) similarly documented male predominance [104]. The 1999–2005 national series reported 56% male [8].

This disparity is most plausibly explained by behavioral exposure factors—males in relevant age groups disproportionately engage in hunting, forestry, farming, and other outdoor activities that increase tick contact in endemic habitats [183]. A One Health occupational health lens is therefore appropriate: forestry workers, farmers, hunters, wildlife biologists, and outdoor maintenance workers in endemic areas represent occupationally at-risk populations warranting targeted tick prevention education and potentially heightened POWV surveillance [183]. Because these outdoor exposures overlap with those for other common tick-borne diseases, public-facing prevention resources emphasize shared tick-avoidance awareness across these infections [184].

### 7.2. Symptom Profile and Female-Specific Considerations

The published literature does not document systematic sex-based differences in POWV symptom character—the neurological syndrome of encephalitis, seizures, aphasia, and paralysis appears consistent across sexes [85]. Published female cases include a 79-year-old Connecticut woman with worsening mental status, weakness, myalgia, fever, and headache whose MRI was initially misread as HSV encephalitis [89]. The female-limited case literature is insufficient to draw definitive conclusions about female-specific presentations.

Pregnancy represents a specific concern warranting attention under the One Health framework. Arboviral infections—including Zika and West Nile virus—carry increased risk of severe maternal and fetal outcomes in pregnant women [185]. No dedicated POWV studies in pregnancy exist, but the potential for neuroinvasive POWV to cause maternal encephalitis or fetal neurological injury warrants heightened clinical vigilance for pregnant patients in endemic areas presenting with unexplained febrile illness or neurological symptoms [1,185].

Kapoor and colleagues documented higher POWV seropositivity rates among individuals with a prior Lyme disease history compared to community controls in the northeastern United States, suggesting that a Lyme disease history may serve as a marker of elevated POWV co-exposure risk warranting heightened clinical awareness [186].

## 8. Powassan Virus Symptoms Compared to Other Tick-Borne Diseases

A central clinical challenge is that POWV’s early symptom profile overlaps substantially with those of other tick-borne diseases circulating in the same geographic regions. This overlap is not incidental but ecologically fundamental: *I. scapularis* co-transmits multiple pathogens—*Borrelia burgdorferi*, *Anaplasma phagocytophilum*, *Babesia microti*, *Borrelia miyamotoi*, and POWV—within the same tick–wildlife–habitat system, and field studies have documented coinfection rates of 10–25% for two or more of these agents in individual ticks from highly endemic areas [50,51,52,53,54]. This shared ecology means that a patient’s tick bite may deliver multiple pathogens simultaneously, that wildlife reservoir hosts sustain transmission cycles for all of these agents concurrently, and that the ecological drivers of POWV risk are inseparable from the ecological drivers of Lyme disease, anaplasmosis, and babesiosis risk. One Health surveillance that monitors the tick-borne disease complex as a system—rather than individual pathogens in isolation—is therefore not merely a methodological preference but an epidemiological necessity [49].

POWV’s cardinal symptom fingerprint begins with a prodrome shared by all tick-borne diseases (fever, headache, myalgia, fatigue) but rapidly evolves to acute severe neurological involvement—a trajectory that is largely absent or greatly delayed in bacterial tick-borne pathogens and distinguishes POWV from every other common U.S. tick-borne disease [187]. POWV encephalitis produces altered mental status, seizures, expressive aphasia, cranial nerve palsies, focal paralysis, and ataxia—a CNS syndrome of greater severity and acuity than Lyme disease, anaplasmosis, babesiosis, ehrlichiosis, or Rocky Mountain spotted fever (RMSF) [30]. Fatal outcomes occur in 10–15% of neuroinvasive POWV cases, and 50% of survivors carry lasting neurological deficits that categorically exceed those of appropriately treated bacterial tick-borne diseases [9]. Powassan encephalitis and Colorado tick fever represent the two tick-transmitted viral encephalitides indigenous to North America; POWV exhibits markedly higher mortality and neurological sequelae than the relatively benign Colorado tick fever, making clinical distinction consequential for prognosis counseling [188].

Three key symptom-based differential markers distinguish POWV from bacterial tick-borne diseases. First, POWV produces no characteristic rash: Lyme disease produces the erythema migrans bull’s-eye rash in 70–80% of early cases, RMSF produces a petechial/purpuric rash spreading centrally from the wrists and ankles, and ehrlichiosis produces a maculopapular rash in approximately 30% of cases [9,187,189]. The absence of any rash in the setting of acute neurological decline should strongly raise suspicion for POWV or another viral etiology. Second, POWV does not produce the leukopenia, thrombocytopenia, and hepatic transaminase elevations that characterize anaplasmosis and ehrlichiosis [190]. Third, and most critical for patient management, POWV does not respond to antibiotic therapy. Lyme disease, anaplasmosis, ehrlichiosis, and RMSF all respond to doxycycline; failure to improve on empirical doxycycline in a patient with tick exposure and encephalitis is a red flag for POWV [190].

POWV also differs from Lyme disease in its transmission kinetics. POWV transmits within 15 min of tick attachment versus 36–48 h for *Borrelia burgdorferi* [22,191]. A patient who promptly removed a tick can still develop POWV encephalitis. Lyme neuroborreliosis presents as an indolent syndrome of facial palsy, radiculopathy, or lymphocytic meningitis over weeks to months—a distinctly different tempo from POWV’s acute meningoencephalitis [92]. When compared to *Borrelia miyamotoi*, which causes relapsing fever with chills and joint pain, POWV lacks the characteristic relapsing fever course [192]. The One Health implication of these comparisons is straightforward: because all of these diseases are transmitted by ticks in the same habitats, they form an ecological cluster that requires integrated surveillance, combined prevention messaging, and comprehensive clinical awareness [49].

Reller and colleagues documented the co-circulation of POWV with anaplasmosis, ehrlichiosis, and Lyme disease in overlapping geographic zones across the upper Midwest and Northeast, emphasizing that clinicians in these regions must maintain broad tick-borne disease differential diagnoses [99]. Serologic surveillance has documented POWV seropositivity in patients initially presenting for suspected Lyme disease evaluation, with detectable POWV IgM in a subset from Lyme-endemic areas, with implications for clinical evaluation algorithms [193]. Microbiome analyses of *I. scapularis* ticks from New York and Connecticut confirm polymicrobial co-carriage of POWV, *B. burgdorferi*, *A. phagocytophilum*, and *B. microti*, directly quantifying co-exposure risk [52,53,194,195]. Knox and colleagues specifically documented POWV/DTV and *B. burgdorferi* co-infection in Wisconsin tick populations, confirming that co-transmission risk extends to the Great Lakes endemic zone [196]. Active tick surveillance in Massachusetts *I. scapularis* populations demonstrated stable POWV infection rates over time and common co-infection with Lyme spirochetes, reinforcing the argument for POWV inclusion in routine tick-borne disease differential panels [65,66]. Active New York State tick surveillance confirmed POWV in *I. scapularis* alongside multiple co-circulating pathogens, supporting county-level risk stratification as a guide for clinical awareness campaigns [197]. Campagnolo and colleagues provided evidence of POWV/DTV in adult black-legged ticks from Pennsylvania, extending the geographic evidence base for POWV co-circulation with Lyme disease into the mid-Atlantic [57]. Human neutralizing antibodies against tick-borne flaviviruses including POWV have been detected in populations from Mexico and Brazil, suggesting cross-reactive immunity that may modulate clinical severity and complicate serodiagnosis in internationally mobile patients [198]. The 2023 nationally notifiable arboviral disease report documented POWV case counts, state distribution, and demographic patterns, providing comprehensive recent epidemiological description of POWV disease burden [142].

The neurological manifestations of these diseases differ markedly in tempo, distribution, and reversibility. POWV produces an acute, often fulminant meningoencephalitis—altered consciousness, seizures, expressive aphasia, cranial neuropathies, focal paralysis, cerebellar ataxia, and brainstem rhombencephalitis—with high mortality and frequent permanent deficits. Lyme neuroborreliosis, in contrast, is subacute to chronic and characteristically causes cranial neuritis (notably facial-nerve palsy, sometimes bilateral), painful lymphocytic meningitis, and radiculoneuritis (Bannwarth syndrome); parenchymal encephalomyelitis is rare and outcomes are generally good after antibiotics. Neurologic involvement in anaplasmosis is uncommon and usually mild—reported as occasional meningoencephalitis, brachial plexopathy, or demyelinating polyneuropathy, typically with normal or only minimally abnormal CSF and resolution on doxycycline. Consequently, an acute, rapidly progressive encephalitis with prominent cortical and brainstem signs that fails to respond to empirical antibiotics points to POWV rather than to neuroborreliosis or anaplasmosis [92,99,100].

Beyond diagnostic overlap, co-infection carries specific clinical implications that bear directly on management. Because *I. scapularis* can transmit *Borrelia burgdorferi*, *Anaplasma phagocytophilum*, and *Babesia microti* in a single bite, a patient may present with simultaneous infections whose treatable bacterial or protozoal components—responsive to doxycycline or atovaquone–azithromycin—coexist with untreatable POWV, so that a favorable early response to empirical antibiotics can create false reassurance while neuroinvasive POWV progresses unchecked [50,99]. Co-infection may also modify disease severity: concurrent babesiosis or anaplasmosis can amplify systemic inflammation and complicate interpretation of the thrombocytopenia and transaminitis expected in the bacterial and protozoal infections but not in POWV, obscuring the point at which a deteriorating course reflects central-nervous-system flaviviral disease rather than incompletely treated bacterial illness. These considerations argue for keeping POWV on the differential in any patient from an endemic area who develops encephalitic features despite appropriate antibacterial therapy, and for testing broadly rather than anchoring on the first pathogen identified [99,100].

Table 2 presents a systematic symptom-by-symptom comparison of POWV against the principal U.S. tick-borne diseases; neurological features are grouped under CNS disease and in-text references have been removed for readability.

## 9. Clinical Management

No specific antiviral therapy is currently approved for POWV disease—a critical therapeutic gap that underscores the urgency of prevention [55]. This absence of a specific treatment is itself a One Health call to action: the same investment in wildlife and environmental surveillance that could enable earlier human risk detection could also feed into vaccine development timelines and antiviral drug target identification [199].

Management is entirely supportive. Non-neuroinvasive POWV illness is managed with rest, hydration, antipyretics, and close monitoring for CNS progression [200]. Neuroinvasive POWV disease typically requires hospitalization; 91% of New York State cases required admission [104]. Patients with encephalitis require neurological monitoring for elevated intracranial pressure, seizures, and respiratory compromise. Osmotherapy (hypertonic saline or mannitol), intracranial pressure monitoring, antiepileptic drugs (e.g., levetiracetam), and mechanical ventilation may all be required in severe cases [104,201]. Hyponatremia from SIADH requires careful correction.

Corticosteroids and intravenous immunoglobulin (IVIG) have been used as adjunctive therapies in individual case reports, but no clinical trials support their use and both remain off-label [14]. Ribavirin and other flaviviral antivirals have theoretical rationale but no clinical evidence for POWV [85]. Blood donation should be deferred for four months following POWV illness given the documented risk of transfusion-transmitted infection [55].

A comprehensive review of tick-borne disease diagnostic and treatment pathways confirms that POWV-specific protocols for blood product donor screening remain underdeveloped relative to the documented transfusion transmission risk, representing a regulatory gap requiring attention [95].

### Strategies to Improve POWV Diagnosis

The diagnostic gap described above—reliance on send-out serology with slow, cross-reactive results and confirmatory PRNT that requires weeks—invites several concrete improvements. First, incorporating POWV into the multiplex tick-borne disease panels already used for *Borrelia*, *Anaplasma*, *Ehrlichia*, and *Babesia* would ensure that the virus is tested for whenever a tick-borne illness is suspected, rather than only when a clinician specifically requests it. Second, unbiased metagenomic next-generation sequencing (mNGS) of cerebrospinal fluid can detect POWV RNA in unexplained encephalitis without a priori suspicion of the virus, and wider use of mNGS in neurology and infectious-disease practice would capture cases that targeted testing misses [96]. Third, the development and validation of rapid molecular or antigen-based point-of-care assays—analogous to those deployed for other arboviruses—would shorten the time to diagnosis, support earlier supportive management and blood-product deferral, and improve the case ascertainment on which surveillance depends. Because POWV viremia is brief, RT-PCR is most useful very early in illness; expanded IgM-capture assays and standardized reference-laboratory PRNT capacity therefore remain necessary to confirm later-presenting neuroinvasive cases [80,84].

Survivors with neurological sequelae require multidisciplinary rehabilitation: physical therapy for weakness and gait abnormalities, occupational therapy for activities of daily living, speech-language pathology for aphasia, and neuropsychological support for cognitive deficits [201]. The One Health framework highlights that rehabilitation burden extends beyond the patient to families, caregivers, and healthcare systems in endemic communities—a cost borne disproportionately in rural and economically marginalized regions where POWV risk is highest [98].

## 10. State-Level Surveillance Reporting of Powassan Virus: A Review of Forty-Eight State Dashboards

A critical and often overlooked dimension of POWV’s public health profile is the stark heterogeneity of state-level surveillance and data reporting. Unlike federally notifiable diseases that are uniformly tracked through ArboNET and published in annual MMWR summaries, the availability of state-specific POWV data—broken down by case count, geography, demographics, and time trend—varies dramatically from state to state. This variation has direct consequences for research, clinical awareness, and public health response: states that do not report POWV data may fail to recognize emerging local transmission, and clinicians in those states may not receive the targeted alerts that state health surveillance data typically generate.

As part of this scoping review, the publicly accessible tick-borne disease surveillance resources of forty-eight states were examined to determine which states publish quantitative Powassan virus statistics—defined here as case counts or incidence rates resolved by year, county or region, or demographic group—rather than a purely descriptive overview of the disease. Applying this strict criterion, seven states were found to publish POWV statistics: Connecticut, Maine, Massachusetts, Minnesota, New Jersey, New York, and Wisconsin. Table 3 summarizes these seven statistics-providing states, including the years and geographic granularity of the data, the reporting format, the demographic or secondary data available, and a direct link to each state’s primary data source. This represents a deliberately conservative standard: many additional states acknowledge POWV or report isolated cases, but only these seven make structured, quantitative POWV data publicly available.

Among the seven statistics-providing states, the depth and format of reporting vary considerably. Minnesota offers the most comprehensive state-level POWV data, reporting case counts by public health district and year across the period 2008–2022 (with no cases in 2014 or 2015), supplemented by demographic breakdowns (age, sex, month of acquisition), clinical data (diagnostics, outcomes, symptoms), and geographic case location for a subset of years. This level of reporting is enabled by the Minnesota Disease Control Newsletter, a longstanding Department of Health publication that aggregates surveillance data across tick-borne diseases, and it represents a model for what state-level POWV surveillance can achieve. New Jersey and New York are notable for presenting POWV data as interactive, regularly updated resources: New Jersey’s vector-borne disease dashboard reports both case counts and incidence rates alongside tick and mosquito pathogen data, and New York’s annual Tick-Borne Disease Surveillance Report pairs regional case counts and incidence with an open-data tick-infectivity portal on Health Data NY. Maine and Massachusetts maintain dedicated POWV data products—Maine through case records extending to 2000 with county and outcome detail, and Massachusetts through a standalone Powassan surveillance summary and a ten-year tick-borne disease morbidity report current to 2026. Wisconsin, which recorded a state-record twelve cases in 2024, publishes annual case counts chiefly through Department of Health Services advisories rather than a standing dashboard, but its quantitative reporting nonetheless meets the threshold applied here. Connecticut, where POWV disease is a reportable condition, publishes quantitative case data through its Infectious Diseases Annual Statistics Archive rather than a dedicated POWV product: statewide reportable-disease counts are tabulated by year (extending back to the 1980s and current through 2023) and resolved to the county level for the years 2006–2020, satisfying the year-by-year and geographic resolution required here even though the data are embedded within broader infectious-disease tables rather than a standalone Powassan page.

A larger group of states was excluded from Table 3 because, on review, they did not publish quantitative POWV statistics. Several states that might be assumed to report POWV in fact do not: a dedicated POWV data page could not be located for Arkansas or Kansas, and national ArboNET tallies record no recent cases in either state. Other states acknowledge POWV but stop short of structured statistics. Vermont, for example, names its small number of POWV cases (1999, 2022, and 2023) only in narrative text, and its interactive tick-borne disease dashboard is limited to Lyme disease, anaplasmosis, and babesiosis [202]. New Hampshire reports tick-borne disease counts in a periodic infectious disease summary but has noted that its POWV surveillance is incomplete. Maryland’s reporting is event-driven rather than statistical: the state issued a single 2023 press release documenting its first travel-related POWV case and associated death—important clinical communication, but distinct from systematic surveillance. Still other states, including Michigan, North Carolina, North Dakota, Ohio, Rhode Island, Virginia, and West Virginia, have at various points published POWV case numbers within broader tick-borne or arboviral disease reports, but these were dated, intermittent, or embedded as text without the year-by-year, geographic, or demographic resolution required to meet the criterion applied here.

The states that acknowledge POWV without publishing quantitative data are of particular concern from a One Health surveillance perspective, especially where transmission risk is documented. Tennessee, for instance, has confirmed human POWV cases in the peer-reviewed literature yet lists the virus only under emerging diseases without case data, exemplifying the gap between documented disease burden and public surveillance output. A number of states formally classify POWV disease as a reportable or emerging condition—triggering, in principle, mandatory case reporting—while generating no publicly accessible surveillance data. This pattern suggests that the first step toward improving POWV surveillance in many states is not technical capacity but administrative classification and public data release.

From a One Health perspective, the absence of robust state-level POWV surveillance in most U.S. states creates a self-reinforcing cycle: clinicians unaware of local POWV activity order fewer diagnostic tests; fewer confirmed cases are identified; state health departments lack the case volume to justify surveillance investment; and the published case count underestimates the true burden, further dampening clinical and public health urgency. Breaking this cycle requires the proactive state-level designation of POWV disease as a reportable condition, investment in accessible diagnostic testing through clinical laboratories (rather than only public health laboratories), and the integration of POWV reporting into the existing tick-borne disease dashboards that already track Lyme disease, anaplasmosis, and babesiosis in endemic states.

### Public Health Priorities

Synthesizing the evidence above, we highlight five actionable public-health priorities for POWV. (1) Integrate surveillance across sectors, linking human case reporting with entomological monitoring of *I. scapularis* infection prevalence and wildlife-sentinel serosurveys, so that rising viral prevalence in deer or ticks—as was observed in Connecticut deer before the rise in human cases—can trigger clinician alerts and prevention campaigns before people are diagnosed. (2) Establish periodic population-based human serosurveillance to define true endemicity and correct the undercount inherent in passive case reporting. (3) Standardize and expand state-level reporting so that POWV case counts are published with geographic and demographic resolution, breaking the self-reinforcing cycle in which low awareness begets low testing and apparently low burden. (4) Close the diagnostic gap by adding POWV to multiplex tick-borne panels, expanding metagenomic sequencing for unexplained encephalitis, and developing point-of-care assays. (5) Invest in prevention and countermeasures—community-level tick and deer management at the wildland–urban interface, public messaging that tick checks alone are insufficient given the short transmission window, and cross-sectoral support for POWV/TBE vaccine development. As a concrete model, a One Health surveillance program might pair a state health department’s human case data with academic tick-drag surveys and state wildlife-agency deer-serology sampling on a shared data platform, so that entomological and veterinary early-warning signals are acted upon within the human health system rather than remaining siloed.

## 11. Limitations

The following limitations are applicable to this study, largely reflecting limitations to POWV surveillance in general. First, the total number of documented U.S. POWV cases remains small, limiting statistical power for analyses of symptom associations by age, sex, or geography. Second, publication bias substantially overrepresents severe neurological presentations; mild and non-neuroinvasive cases are systematically underrepresented in the extant literature. Third, surveillance underreporting—driven by low clinician awareness, restricted diagnostic testing access, and incomplete case ascertainment—obscures the true POWV disease burden. Fourth, systematic data on sex-specific symptom differences are sparse, and the female case literature is too limited for definitive conclusions. Fifth, racial and ethnic data are inconsistently reported; the New York State analysis found most cases in White, non-Hispanic individuals, but whether this reflects differential exposure, healthcare access, or biological susceptibility is unknown. Sixth, longitudinal follow-up data on POWV survivor outcomes are scarce, and the 50% sequelae figure is based on a relatively short-term follow-up. Finally, from a One Health perspective, integrated human–animal–environmental surveillance data are largely unavailable in published form, limiting access to evidence on the depth to which ecological drivers can be linked to human case patterns in this review. An additional methodological consideration concerns the AI-integrative approach itself: while Claude (Anthropic) substantially accelerated synthesis and cross-referencing across a large and heterogeneous body of sources, the completeness and accuracy of the resulting evidence base depended fundamentally on the curated inputs supplied to it—most importantly the GIDEON database and human-retrieved surveillance records. AI synthesis operating on its training corpus alone would have omitted the most recent case reports, state surveillance updates, and database-aggregated epidemiological figures incorporated here; conversely, the database alone would not have surfaced or contextualized the full primary literature. This interdependence is a limitation only insofar as it underscores that AI-assisted reviews cannot be treated as self-sufficient and must be paired with authoritative data sources and expert human curation to be complete and reliable.

## 12. Conclusions

Powassan virus is, at its core, a One Health phenomenon: its presence in humans depends on an enzootic cycle sustained by wildlife reservoir communities and by tick vectors whose range and abundance are shaped by climate and land use, and it is embedded in a broader complex of co-circulating tick-borne pathogens. The cases clinicians diagnose are the visible tip of a much larger ecological iceberg, so addressing POWV requires intervening across the whole system rather than only at the point of clinical presentation [203,204,205].

The northeastern and Great Lakes states remain the endemic core, but viral evidence in ticks, wildlife, and human cases from the mid-Atlantic, southern, and western United States shows that the risk is not geographically bounded; clinicians in all regions should consider POWV in unexplained acute encephalitis, particularly with relevant travel. Older adults bear the greatest severity and mortality—driven in part by immunosenescence—while children, including infants, are not spared. POWV is distinguished from other tick-borne diseases by its flaviviral etiology, ~15-minute transmission window, absence of a characteristic rash, antibiotic resistance, and the severity and acuity of its CNS disease.

With no specific antiviral or approved vaccine, prevention is the cornerstone. Because transmission can occur within 15 min, tick checks alone are insufficient and must be paired with EPA-registered repellents, protective clothing, and yard management [34,183,206]; upstream ecological measures—reducing the deer density, removing invasive Japanese barberry, and host-targeted acaricide application—can reduce local tick abundance, complementing individual protective measures [17,26,33].

A genuine One Health response also requires integrated surveillance that links human clinical reporting, periodic population-based serosurveillance, entomological monitoring of *I. scapularis* and POWV/DTV prevalence, wildlife sentinels, and ecological data into a real-time, spatially explicit risk index—wildlife signals such as rising deer seroprevalence have historically preceded human cases [1,44,137]. Vaccine development is constrained by small, geographically concentrated case numbers and by lower immunogenicity in older adults, but cross-protective tick-borne encephalitis serocomplex, virus-like-particle, and mRNA approaches, together with immunobridging to licensed TBE vaccines, offer plausible paths forward [1,199].

Methodologically, this review also models transparent human–AI–GIDEON collaboration: of its 206 references, 115 derived from the GIDEON database and 91 from human–AI synthesis of the broader literature and surveillance record, with interpretation and final wording under human control—neither stream alone yielded a complete account of POWV in the United States [43,74,75].

### Future Directions

Priorities for future work include: (1) prospective, geographically stratified human serosurveys to define true endemicity and correct the passive-surveillance undercount; (2) inclusion of POWV in multiplex tick-borne and cerebrospinal-fluid metagenomic panels and the development of validated point-of-care assays; (3) integrated human–animal–environmental surveillance with wildlife and entomological early-warning triggers; (4) studies of how coinfection and host factors such as immunosenescence and anti–type I interferon autoantibodies affect neuroinvasive outcomes; and (5) advancement of POWV and TBE-serocomplex vaccines and candidate antivirals through cross-sectoral funding and adapted regulatory pathways [1,199].

## Figures and Tables

**Table 1:** State-level epidemiological characteristics of Powassan virus disease in the principal reporting states. Case totals are drawn from CDC ArboNET and state health-department reports for the periods indicated; localities reflect counties with documented recurrent activity.

State	Cumulative Cases (Period)	Highest-Burden Localities	Deaths on Record	Distinctive Epidemiological Feature
New York	44 (2013–2023)	Columbia & Putnam counties (Hudson Valley); Albany Capital Region	≥1	Most detailed county-level data; cases cluster at the suburban–forest interface
Minnesota	32 (2008–2017); 14 in 2024 (national leader)	Crow Wing, Aitkin, Cass, Hubbard; Winona, Olmsted	≥4 (2011, 2021 × 2, 2022)	Most comprehensive state reporting; consistently leads the nation in counts
Wisconsin	68 (2003–2024); 12 in 2024 (state record)	Vilas, Oneida, Forest, Lincoln, Price (northern forested counties)	≥1	Site of the only U.S. transfusion-transmitted POWV case
Maine	≥25 since 2014; record 7 in 2023	York, Cumberland, Sagadahoc, Kennebec, Lincoln	4 since 2015	Highest per-capita incidence nationally (0.50/100,000 in 2023)
Massachusetts	16 (2008–2017)	Boston fringe, North Shore, western MA (e.g., Sharon)	—	“Northeastern epicenter”; source of key clinical case series
Connecticut	Recurrent annual cases (2016–2023)	Statewide; tick infection up to 4.2%	—	Documented brief-attachment transmission; recurrent yearly activity
New Jersey	7 (2008–2017; 5th nationally)	Sussex County (cluster of 3 in 2019)	≥1	Serosurvey estimated ~23% of infections are neuroinvasive

**Table 2:** Symptom and clinical feature comparison: Powassan virus versus common U.S. tick-borne diseases. Neurological manifestations are grouped under a single CNS disease row, and supporting references (cited in the text) have been removed for clarity. RMSF = Rocky Mountain spotted fever; *B. miyamotoi* = *Borrelia miyamotoi*; CN = cranial nerve.

Feature	Powassan Virus	Lyme Disease	Anaplasmosis	Babesiosis	Ehrlichiosis	RMSF	*B. miyamotoi*
Fever	Acute, high-grade; near-universal	Common; early	Prominent	Prominent	Prominent	Prominent	Yes; relapsing
Headache	Severe; persistent	Mild–moderate; early	Prominent	Present	Present	Severe	Present
Myalgia/fatigue	Yes; prodromal	Prominent; early	Prominent	Prominent	Prominent	Prominent	Prominent
Characteristic rash	None (rare maculopapular)	Erythema migrans (bull’s-eye) 70–80%	Rare	None	~30% maculopapular	Petechial/purpuric	None
CNS disease (encephalitis, meningitis, altered mental status, seizures, aphasia, paralysis, cranial neuropathy, ataxia, rhombencephalitis)	Acute, severe, predominant; full syndrome including seizures, aphasia, paralysis, cranial palsies, ataxia, and brainstem involvement	Neuroborreliosis: subacute cranial (CN VII) palsy, lymphocytic meningitis, radiculopathy	Rare; usually mild	Rare (severe/immunocompromised)	Rare; occasional meningoencephalitis	Possible in severe disease	Rare; meningoencephalitis in immunocompromised
Long-term neurological sequelae	~50% of survivors	Rare (treated)	Rare	Rare	Rare	Possible (untreated)	Rare
Joint/bone pain	Absent	Prominent (late arthritis)	Rare	Rare	Rare	Rare	Yes
Hemolytic anemia	No	No	No	Hallmark	No	No	No
Leukopenia/thrombocytopenia	Not characteristic	No	Hallmark	Thrombocytopenia	Hallmark	Thrombocytopenia	No
Elevated hepatic transaminases	Not characteristic	No	Common	Possible	Common	Possible	No
Responds to antibiotics	No (viral)	Yes; doxycycline	Yes; doxycycline	Yes; atovaquone	Yes; doxycycline	Yes; doxycycline	Yes; doxycycline
Transmission window	≥15 min attachment	36–48 h attachment	Hours	Hours	Hours	Hours	Hours
Case fatality rate	10–15% (neuroinvasive)	<1% (treated)	Rare (treated)	<1% (treated)	Rare (treated)	5–10% (treated)	Rare

**Table 3:** States publishing quantitative Powassan virus statistics: data years, geographic scope, format, secondary data, and sources.

State	Data Years/Coverage	Geographic Scope	Reporting Format	Secondary and Demographic Data	Primary Source	Data Gaps/Limitations
Connecticut	Reportable-disease counts by year (1980s–2023); by-county 2006–2020	Statewide and by-county (2006–2020)	Infectious Diseases Annual Statistics Archive (PDF tables)	Annual case counts; county counts (2006–2020); reportable-disease totals	CT DPH	County-level detail ends 2020; embedded in broader infectious-disease tables rather than a standalone POWV product
Maine	Cases tracked since 2000; ≥25 catalogued 2014–2025	Statewide and by-county, by year	Webpage; PDF/HTML press releases; data reports	Sex; age; county; deaths; onset; outcomes	Maine PH	Case counts largely via press releases/reports rather than a standing dashboard; no published incidence rates
Massachusetts	Surveillance summary 2013–2018; morbidity report current to 2026	Statewide annual counts	Dedicated POWV summary document; 10-year tick-borne disease report	Month of onset; symptoms (2013–2018); annual counts	MA DPH	Detailed symptom/demographic data limited to 2013–2018; recent years reported as annual counts only
Minnesota	2008–2022 by public health district (no cases in 2014 or 2015)	By public health district and year	Statistics webpage; PDF graph; Disease Control Newsletter	Age; sex; month; diagnostics; outcomes; symptoms (select years)	MN Dept. of Health	No cases reported for 2014–2015; sub-district geographic resolution and case-level incidence rates not published
New Jersey	Multi-year statewide and by-county; weekly in-season reports	Statewide and by-county (most granular)	Interactive vector-borne disease dashboard; weekly surveillance reports	Case counts; incidence rates; sex; age group; tick/mosquito pathogens; ED visits	NJ DOH	Among the most complete; principal gap is reconciling weekly in-season counts with final annual totals
New York	Cases by region 2024; incidence rates 2020–2024; tick infectivity 2015–2024	By region; tick infectivity by county	Tick-borne disease PDF report; Health Data NY open-data portal	Incidence by region; entomologic risk index; tick infectivity	Health Data NY	Regional (not county-level) case resolution; incidence emphasized over absolute counts in some products
Wisconsin	Annual counts via DHS advisories (12 cases in 2024, a state record)	Statewide only	POWV disease webpage; DHS health advisories and bulletins	Annual case counts; limited demographics	WI DHS	Statewide only—no county or demographic breakdown; counts conveyed through advisories rather than a data product

Note: Table includes only states that publish quantitative POWV data (case counts or incidence rates resolved by year, geography, or demographic group). States that list POWV disease as a notifiable or emerging condition, mention it in a descriptive overview, or report only isolated press-release cases without structured data were excluded and discussed in the text. Connecticut’s POWV counts appear within its Infectious Diseases Annual Statistics Archive (CT DPH): https://portal.ct.gov/dph/infectious-diseases/id-stats-archive (accessed on 15 June 2026). National POWV data are available from CDC ArboNET: https://www.cdc.gov/powassan/data-maps/historic-data.html (accessed on 15 June 2026) [11].

## Data Availability

All data supporting the findings of this review are available in the cited published literature, including the GIDEON Infectious Diseases Database (subscription required) and the state-level tickborne disease surveillance resources linked in Table 3. State-level surveillance data were sourced by the primary author over a six-year period through official public health portals, local library archival retrieval, and direct access to state health department publications; these materials were provided to the AI partner (Claude, Anthropic) for synthesis and integration. No new primary datasets were generated. Researchers seeking to replicate the AI-integrative methodology are directed to the Methods section, which describes the three-way partnership among the human investigators, Claude, and GIDEON.

## Supplementary Materials

The following supporting information can be downloaded at https://www.mdpi.com/article/10.3390/idr18050096/s1, Table S1: Preferred Reporting Items for Systematic reviews and Meta-Analyses extension for Scoping Reviews (PRISMA-ScR) Checklist.

## References

[1] Kemenesi G., Banyai K. (2019). Tick-borne Flaviviruses, with a focus on Powassan Virus. Clin. Microbiol. Rev..

[2] Ebel G.D. (2010). Update on Powassan virus: Emergence of a North American tick-borne flavivirus. Annu. Rev. Entomol..

[3] Centers for Disease Control and Prevention (2026). About Powassan Virus. https://www.cdc.gov/powassan/about/index.html.

[4] Gibbs E.P.J. (2014). The evolution of One Health: A decade of progress and challenges for the future. Vet. Rec..

[5] Campbell O., Krause P.J. (2020). The emergence of human Powassan virus infection in North America. Ticks Tick Borne Dis..

[6] McLean D.M., Donohue W.L. (1959). Powassan virus: Isolation of virus from a fatal case of encephalitis. Can. Med. Assoc. J..

[7] Thomas L.A., Kennedy R.C., Eklund C.M. (1960). Isolation of a virus closely related to Powassan virus from *Dermacentor andersonii* collected along the Cache la Poudre River, Colorado. Proc. Soc. Exp. Biol. Med..

[8] Hinten S.R., Beckett G.A., Gensheimer K.F., Pritchard E., Courtney T.M., Sears S.D., Woytowicz J.M., Preston D.G., Smith J.R.P., Rand P.W. (2008). Increased recognition of Powassan encephalitis in the United States, 1999–2005. Vector Borne Zoonotic Dis..

[9] Krow-Lucal E.R., Lindsey N.P., Fischer M., Hills S.L. (2018). Powassan virus disease in the United States, 2006–2016. Vector Borne Zoonotic Dis..

[10] Center for Infectious Disease Research and Policy (CIDRAP) Surveillance Data Show Rise in US Powassan Virus Cases. University of Minnesota, 2024. https://www.cidrap.umn.edu/tick-borne-disease/surveillance-data-show-rise-us-powassan-virus-cases.

[11] Centers for Disease Control and Prevention Historic Data (2004–2024): Powassan Virus. [ArboNET Interactive Map; Cases by State and Year, 2004–2024; Data Finalized 24 February 2026]. https://www.cdc.gov/powassan/data-maps/historic-data.html.

[12] Hassett E.M., Thangamani S. (2021). Ecology of Powassan Virus in the United States. Microorganisms.

[13] Vogels C.B.F., Brackney D.E., Dupuis A.P., Robich R.M., Fauver J.R., Brito A.F., Williams S.C., Anderson J.F., Lubelczyk C.B., Lange R.E. (2023). Phylogeographic reconstruction of the emergence and spread of Powassan virus in the northeastern United States. Proc. Natl. Acad. Sci. USA.

[14] Kakoullis L., Vaz V.R., Kaur D., Kakoulli S., Panos G., Chen L.H., Behlau I. (2023). Powassan virus infections: A systematic review. Trop. Med. Infect. Dis..

[15] Maxwell S.P., Brooks C., Kim P., Kim D., McNeely C.L., Thomas K. (2023). Understanding Habitats and Environmental Conditions of White-Tailed Deer Population Density and Public Health Data to Aid in Assessing Human Tick-Borne Disease Risk. Microorganisms.

[16] Anis H., Basha Shaik A., Karabulut E., Uzun M., Tiwari A., Nazir A., Alemayehu A. (2023). Upsurge of Powassan virus disease in northeastern United States: A public health concern. Ann. Med. Surg..

[17] Dash C.K., Rothman R., Gill A., Bhanot N., Min Z. (2025). An Emerging Cause of Arboviral Encephalitis in Pennsylvania. Cureus.

[18] Piantadosi A., Solomon I.H. (2022). Powassan virus encephalitis. Infect. Dis. Clin. N. Am..

[19] Padda H., Huang C.Y.-H., Grimm K., Biggerstaff B.J., Ledermann J.P., Raetz J., Boroughs K., Mossel E.C., Martin S.W., Lehman J.A. (2025). Powassan and Eastern Equine Encephalitis Virus Seroprevalence in Endemic Areas, United States, 2019–2020. Emerg. Infect. Dis..

[20] Smith R.P., Elias S.P., Cavanaugh C.E., Lubelczyk C.B., Lacombe E.H., Brancato J., Doyle H., Rand P.W., Ebel G.D., Krause P.J. (2019). Seroprevalence of *Borrelia burgdorferi*, *B. miyamotoi*, and Powassan Virus in Residents Bitten by *Ixodes* Ticks, Maine, USA. Emerg. Infect. Dis..

[21] Centers for Disease Control and Prevention Symptoms, Diagnosis, and Treatment|Powassan Virus. https://www.cdc.gov/powassan/symptoms-diagnosis-treatment/index.html.

[22] Ebel G.D., Kramer L.D. (2004). Short report: Duration of tick attachment required for transmission of Powassan virus by deer ticks. Am. J. Trop. Med. Hyg..

[23] Hermance M.E., Thangamani S. (2017). Powassan virus: An emerging arbovirus of public health concern in North America. Vector Borne Zoonotic Dis..

[24] Costero A., Grayson M.A. (1996). Experimental transmission of Powassan virus (*Flaviviridae*) by *Ixodes scapularis* ticks (Acari: Ixodidae). Am. J. Trop. Med. Hyg..

[25] Smith R., Woodall J.P., Whitney E., Deibel R., Gross M.A., Smith V., Bast T.F. (1974). Powassan virus infection. Can. Med. Assoc. J..

[26] Stafford K.C. (2007). Tick Management Handbook.

[27] Deardorff E.R., Nofchissey R.A., Cook J.A., Hope A.G., Tsvetkova A., Talbot S.L., Ebel G.D. (2013). Powassan virus in mammals, Alaska and New Mexico, U.S.A.; and Russia, 2004–2007. Emerg. Infect. Dis..

[28] Ebel G.D., Campbell E.N., Goethert H.K., Spielman A., Telford S.R. (2000). Enzootic transmission of deer tick virus in New England and Wisconsin sites. Am. J. Trop. Med. Hyg..

[29] Stafford K.C., Williams S.C. (2014). Deer Tick (Blacklegged Tick).

[30] Yale Medicine Powassan Virus Fact Sheet. https://www.yalemedicine.org/conditions/powassan-virus.

[31] McMinn R.J., Langsjoen R.M., Bombin A., Robich R.M., Ojeda E., Normandin E., Goethert H.K., Lubelczyk C.B., Schneider E., Cosenza D. (2023). Phylodynamics of deer tick virus in North America. Virus Evol..

[32] Deshpande G., Beetch J.E., Heller J.G., Naqvi O.H., Kuhn K.G. (2024). Assessing the influence of climate change and environmental factors on the top tick-borne diseases in the United States: A systematic review. Microorganisms.

[33] Baxter L., Lubelczyk C., Harrington L.C., Angelico J., Meagher M.C., Smith R.P., Robich R.M. (2024). Features of Consistent Powassan Virus Lineage II Focus in Southern Maine, United States. Am. J. Trop. Med. Hyg..

[34] Centers for Disease Control and Prevention (2021). Preventing Tick Bites.

[35] Lessani M.N., Li Z., Jing F., Qiao S., Zhang J., Olatosi B., Li X. (2024). Human mobility and the infectious disease transmission: A systematic review. Geo. Spat. Inf. Sci..

[36] TravelHealthPro. Outbreaks. https://travelhealthpro.org.uk/outbreaks.

[37] StatPearls (2023). Powassan Virus. NCBI Bookshelf.

[38] El Khoury M.Y., Camargo J.F., White J.L., Backenson B.P., Dupuis A.P., Escuyer K.L., Kramer L., George K.S., Chatterjee D., Prusinski M. (2013). Potential role of deer tick virus in Powassan encephalitis cases in Lyme disease-endemic areas of New York, U.S.A. Emerg. Infect. Dis..

[39] Peña Rosado A., Pinto A.K. (2026). Powassan virus. Trends Microbiol..

[40] Normandin E., Solomon I.H., Zamirpour S., Lemieux J., A Freije C., Mukerji S.S., Tomkins-Tinch C., Park D., Sabeti P.C., Piantadosi A. (2020). Powassan virus neuropathology and genomic diversity in patients with fatal encephalitis. Open Forum Infect. Dis..

[41] Mladinich M.C., Himmler G.E., Conde J.N., Gorbunova E.E., Schutt W.R., Sarkar S., Tsirka S.-A.E., Kim H.K., Mackow E.R. (2024). Age-dependent Powassan virus lethality is linked to glial cell activation and divergent neuroinflammatory cytokine responses in a murine model. J. Virol..

[42] Telford S.R., Piantadosi A. (2023). Powassan virus persistence after acute infection. mBio.

[43] Berger S.A. (2005). GIDEON: A comprehensive Web-based resource for geographic medicine. Int. J. Health Geogr..

[44] Nofchissey R.A., Deardorff E.R., Blevins T.M., Anishchenko M., Bosco-Lauth A., Berl E., Lubelczyk C., Mutebi J.-P., Brault A.C., Ebel G.D. (2013). Seroprevalence of Powassan virus in New England deer, 1979-2010. Am. J. Trop. Med. Hyg..

[45] Main A.J., Carey A.B., Downs W.G. (1979). Powassan virus in *Ixodes cookei* and Mustelidae in New England. J. Wildl. Dis..

[46] Goethert H.K., Mather T.N., Johnson R.W., Telford S.R. (2021). Incrimination of shrews as a reservoir for Powassan virus. Commun. Biol..

[47] Pedersen K., Wang E., Weaver S.C., Wolf P.C., Randall A.R., Van Why K.R., Da Rosa A.P.T., Gidlewski T. (2017). Serologic Evidence of Various Arboviruses Detected in White-Tailed Deer (*Odocoileus virginianus*) in the United States. Am. J. Trop. Med. Hyg..

[48] Zarnke R.L., Yuill T.M. (1981). Powassan virus infection in snowshoe hares (*Lepus americanus*). J. Wildl. Dis..

[49] Madison-Antenucci S., Kramer L.D., Gebhardt L.L., Kauffman E. (2020). Emerging tick-borne diseases. Clin. Microbiol. Rev..

[50] Nijjar L.S., Schwartz S., Koon D.S.K., Marin S.M., Jimenez M.E., Williams T., Chinnici N. (2024). Prevalence of *Babesia microti* Co-Infection with Other Tick-Borne Pathogens in Pennsylvania. Microorganisms.

[51] Narvaez Z.E., Rainey T., Puelle R., Khan A., Jordan R.A., Egizi A.M., Price D.C. (2023). Detection of multiple tick-borne pathogens in *Ixodes scapularis* from Hunterdon County, NJ, USA. Curr. Res. Parasitol. Vector Borne Dis..

[52] Schwartz S., Calvente E., Rollinson E., Koon D.S.K., Chinnici N. (2022). Tick-Borne Pathogens in Questing Blacklegged Ticks (Acari: Ixodidae) From Pike County, Pennsylvania. J. Med. Entomol..

[53] Tokarz R., Tagliafierro T., Sameroff S., Cucura D.M., Oleynik A., Che X., Jain K., Lipkin W.I. (2019). Microbiome analysis of *Ixodes scapularis* ticks from New York and Connecticut. Ticks Tick Borne Dis..

[54] Sanchez-Vicente S., Tagliafierro T., Coleman J.L., Benach J.L., Tokarz R. (2019). Polymicrobial Nature of Tick-Borne Diseases. mBio.

[55] Centers for Disease Control and Prevention Treatment and Prevention of Powassan Virus Disease. https://www.cdc.gov/powassan/hcp/treatment-prevention/index.html.

[56] Whitlow A.M., Cumbie A.N., Eastwood G. (2022). Pathogen prevalence in *Amblyomma americanum* and *Ixodes scapularis* ticks from central Appalachian Virginia, U.S.A.. J. Vector Ecol..

[57] Campagnolo E.R., Tewari D., Farone T.S., Livengood J.L., Mason K.L. (2018). Evidence of Powassan/deer tick virus in adult black-legged ticks (*Ixodes scapularis*) recovered from hunter-harvested white-tailed deer (*Odocoileus virginianus*) in Pennsylvania. Zoonoses Public Health.

[58] Corrin T., Greig J., Harding S., Young I., Mascarenhas M., Waddell L.A. (2018). Powassan virus, a scoping review of the global evidence. Zoonoses Public Health.

[59] Johnson H.N. (1987). Isolation of Powassan virus from a spotted skunk in California. J. Wildl. Dis..

[60] Brackney D.E., Nofchissey R.A., Fitzpatrick K.A., Brown I.K., Ebel G.D. (2008). Stable prevalence of Powassan virus in *Ixodes scapularis* in a northern Wisconsin focus. Am. J. Trop. Med. Hyg..

[61] Dupuis A.P., Peters R.J., Prusinski M.A., Falco R.C., Ostfeld R.S., Kramer L.D. (2013). Isolation of deer tick virus (Powassan virus, lineage II) from *Ixodes scapularis* and detection of antibody in vertebrate hosts sampled in the Hudson Valley, New York State. Parasit. Vectors.

[62] Hart C., Hassett E., Vogels C.B., Shapley D., Grubaugh N.D., Thangamani S. (2023). Powassan Virus Lineage I in Field-Collected *Dermacentor variabilis* Ticks, New York, USA. Emerg. Infect. Dis..

[63] Garba A., Riley J., Lahmers K.K., Eastwood G. (2024). Widespread Circulation of Tick-Borne Viruses in Virginia: Evidence of Exposure to Heartland, Bourbon, and Powassan Viruses in Wildlife and Livestock. Microorganisms.

[64] Anderson J.F., Armstrong P.M. (2012). Prevalence and genetic characterization of Powassan virus strains infecting *Ixodes scapularis* in Connecticut. Am. J. Trop. Med. Hyg..

[65] Xu G., Siegel E., Fernandez N., Bechtold E., Daly T., Dupuis A.P., Ciota A., Rich S.M. (2024). Active Surveillance of Powassan Virus in Massachusetts *Ixodes scapularis* Ticks, Comparing Detection Using a New Triplex Real-Time PCR Assay with a Luminex Vector-Borne Panel. Viruses.

[66] Robich R.M., Cosenza D.S., Elias S.P., Henderson E.F., Lubelczyk C.B., Welch M., Smith R.P. (2019). Prevalence and Genetic Characterization of Deer Tick Virus (Powassan Virus, Lineage II) in *Ixodes scapularis* Ticks Collected in Maine. Am. J. Trop. Med. Hyg..

[67] LoGiudice K., Ostfeld R.S., Schmidt K.A., Keesing F. (2003). The ecology of infectious disease: Effects of host diversity and community composition on Lyme disease risk. Proc. Natl. Acad. Sci. USA.

[68] Allan B.F., Keesing F., Ostfeld R.S. (2003). Effect of forest fragmentation on Lyme disease risk. Conserv. Biol..

[69] Ogden N.H., Lindsay L.R., Hanincová K., Barker I.K., Bigras-Poulin M., Charron D.F., Heagy A., Francis C.M., O’Callaghan C.J., Schwartz I. (2008). Role of migratory birds in introduction and range expansion of Ixodes scapularis ticks and of Borrelia burgdorferi and Anaplasma phagocytophilum in Canada. Appl. Environ. Microbiol..

[70] Tricco A.C., Lillie E., Zarin W., O’Brien K.K., Colquhoun H., Levac D., Moher D., Peters M.D.J., Horsley T., Weeks L. (2018). PRISMA Extension for Scoping Reviews (PRISMA-ScR). Ann. Intern. Med..

[71] Arksey H., O’Malley L. (2005). Scoping studies: Towards a methodological framework. Int. J. Soc. Res. Methodol..

[72] Levac D., Colquhoun H., O’Brien K.K. (2010). Scoping studies: Advancing the methodology. Implement. Sci..

[73] Munn Z., Peters M.D.J., Stern C., Tufanaru C., McArthur A., Aromataris E. (2018). Systematic review or scoping review? Guidance for authors when choosing between a systematic or scoping review approach. BMC Med. Res. Methodol..

[74] GIDEON Informatics, Inc. Powassan Virus Disease Profile. In *GIDEON Infectious Diseases Database*; Accessed 2020–2026 via Institutional Subscription. https://app.gideononline.com/.

[75] Sarantopoulos A., Kourmpani C.M., Yokarasa A.L., Makamanzi C., Antoniou P., Spernovasilis N., Tsioutis C. (2024). Artificial intelligence in infectious disease clinical practice: An overview of gaps, opportunities, and limitations. Trop. Med. Infect. Dis..

[76] Villanueva-Miranda I., Xiao G., Xie Y. (2025). Artificial intelligence in early warning systems for infectious disease surveillance: A systematic review. Front. Public Health.

[77] Ali H.H., Ali H.M., A Ali M., Zaky A.F., A Touk A., Darwiche A.H., A Touk A. (2025). The role and limitations of artificial intelligence in combating infectious disease outbreaks. Cureus.

[78] Artsob H., Monath T.P. (1988). Powassan encephalitis. The Arboviruses: Epidemiology and Ecology.

[79] Centers for Disease Control and Prevention Clinical Signs and Symptoms of Powassan Virus Disease. https://www.cdc.gov/powassan/hcp/clinical-signs/index.html.

[80] Bazer D.A., Orwitz M., Koroneos N., Syritsyna O., Wirkowski E. (2022). Powassan encephalitis: A case report from New York, USA. Case Rep. Neurol. Med..

[81] Fareed A., Rohail S., Zameer U., Wahid A., Akhtar S.M.M., Masood W. (2024). A comprehensive neurological perspective on tick-borne flaviviruses, with emphasis on Powassan virus. Ther. Adv. Infect. Dis..

[82] Piantadosi A., Rubin D.B., McQuillen D.P., Hsu L., Lederer P.A., Ashbaugh C.D., Duffalo C., Duncan R., Thon J., Bhattacharyya S. (2016). Emerging Cases of Powassan Virus Encephalitis in New England: Clinical Presentation, Imaging, and Review of the Literature. Clin. Infect. Dis..

[83] Rupani A., Elshabrawy H.A., Bechelli J. (2022). Dermatological manifestations of tick-borne viral infections found in the United States. Virol. J..

[84] Pach J.J., Zubair A.S., Traner C., Falcone G.J., Dewey J.J. (2021). Powassan meningoencephalitis: A case report highlighting diagnosis and management. Cureus.

[85] Ndukwe C., Melville A.C., Osman M., Mohammed Y., Oduro M., Ankrah P.K. (2024). Neurological Complications Associated With the Powassan Virus and Treatment Interventions. Cureus.

[86] Uddin M., Cunha C., Dogon C. (2025). Powassan Virus-Related Encephalitis in the Off-Season: A Case Report. J. Brown Hosp. Med..

[87] Holbrook M.R. (2020). Tick-borne encephalitis. Mandell, Douglas, and Bennett’s Principles and Practice of Infectious Diseases.

[88] Mendoza M.A., Hass R.M., Vaillant J., Johnson D.R., Theel E.S., Toledano M., Abu Saleh O. (2024). Powassan Virus Encephalitis: A Tertiary Center Experience. Clin. Infect. Dis..

[89] Uprety A., Bhusal T., Crespo-Quezada J., Carmona Pires F., Morgan C. (2025). A Case Series of Powassan Meningoencephalitis in a Single Institution in Connecticut with one case resembling Herpes Simplex Virus (HSV) meningoencephalitis on MRI. Cureus.

[90] Minnesota Department of Health Powassan Virus Disease Information for Health Professionals. https://www.health.state.mn.us/diseases/powassan/hcp.html.

[91] Dumic I., Madrid C., Vitorovic D. (2021). Unusual cause at an unusual time—Powassan virus rhombencephalitis. Int. J. Infect. Dis..

[92] Halperin J.J. (2019). Lyme neuroborreliosis. Curr. Opin. Infect. Dis..

[93] Thomm A.M., Schotthoefer A.M., Dupuis A.P., Kramer L.D., Frost H.M., Fritsche T.R., Harrington Y.A., Knox K.K., Kehl S.C. (2018). Development and Validation of a Serologic Test Panel for Detection of Powassan Virus Infection in U.S. Patients Residing in Regions Where Lyme Disease Is Endemic. mSphere.

[94] Centers for Disease Control and Prevention (2025). Clinical Testing and Diagnosis for Powassan Virus Disease. https://www.cdc.gov/powassan/hcp/diagnosis-testing/index.html.

[95] Rowan S., Mohseni N., Chang M., Burger H., Peters M., Mir S. (2023). From Tick to Test: A Comprehensive Review of Tick-Borne Disease Diagnostics and Surveillance Methods in the United States. Life.

[96] Klontz E.H., Solomon I.H., Turbett S.E., Lemieux J.E., Branda J.A. (2024). Cerebrospinal fluid metagenomics has greatest added value as a test for Powassan virus among patients in New England with suspected central nervous system infection. Diagn. Microbiol. Infect. Dis..

[97] Gnanaprakasam R., Wormser G.P., Keller M. (2024). Background seropositivity to Jamestown Canyon virus can lead to diagnostic confusion. Diagn. Microbiol. Infect. Dis..

[98] Lyme Advise Powassan Virus: Symptoms, Treatment, and Long-Term Management. https://www.lymeadvise.com/powassa-virus.

[99] Reller M.E., Clemens E.G., Bakken J.S., Dumler J.S. (2024). Emerging Tick-borne Infections in the Upper Midwest and Northeast United States Among Patients with Suspected Anaplasmosis. Open Forum Infect. Dis..

[100] Biggs H.M., Behravesh C.B., Bradley K.K., Dahlgren F.S., Drexler N.A., Dumler J.S., Folk S.M., Kato C.Y., Lash R.R., Levin M.L. (2016). Diagnosis and management of tickborne rickettsial diseases. MMWR Recomm. Rep..

[101] Johnston A., Yang J., Schiffman E., Galdys A., Fontana L. (2025). Powassan Virus Encephalitis in an Immunocompromised Patient: A Diagnostic Challenge with Case Report and Literature Review. Case Rep. Infect. Dis..

[102] Gervais A., Bastard P., Bizien L., Delifer C., Tiberghien P., Rodrigo C., Trespidi F., Angelini M., Rossini G., Lazzarotto T. (2024). Auto-Abs neutralizing type I IFNs in patients with severe Powassan, Usutu, or Ross River virus disease. J. Exp. Med..

[103] Greenlaw C., MacRae R., Wilson-Murphy M. (2025). Powassan Virus Encephalitis in Pediatric Patients. J. Child Neurol..

[104] Varon A.R., Prusinski M.A., O’cOnnor C., Maffei J.G., Tomaszek L., Stout J., Payne A.F., Dupuis A.P., Ciota A.T., Carson K. (2026). Epidemiology and risk analysis of Powassan virus infection, New York state, USA, 2013–2023. Epidemiol. Infect..

[105] Centers for Disease Control and Prevention (2001). Outbreak of Powassan encephalitis—Maine and Vermont, 1999–2001. MMWR Morb. Mortal. Wkly. Rep..

[106] (2001). Outbreak of Powassan encephalitis—Maine and Vermont, 1999–2001. JAMA.

[107] Fatmi S.S., Zehra R., Carpenter D.O. (2017). Powassan Virus-A New Reemerging Tick-Borne Disease. Front. Public Health.

[108] Wisconsin Department of Health Services Powassan Virus: Wisconsin Data. https://www.dhs.wisconsin.gov/tick/powassan-data.htm.

[109] Mace K.E., Jacobs D., Gould C.V., Staples J.E., Lyons S.L. (2026). West Nile Virus and Other Nationally Notifiable Arboviral Diseases—United States, 2024. MMWR Morb. Mortal. Wkly. Rep..

[110] Birge J., Sonnesyn S. (2012). Powassan virus encephalitis, Minnesota, USA. Emerg. Infect. Dis..

[111] Neitzel D.F., Lynfield R., Smith K. (2013). Powassan virus encephalitis, Minnesota, USA. Emerg. Infect. Dis..

[112] Cary Institute of Ecosystem Studies Black-Legged Ticks Linked to Encephalitis in New York State. https://www.sciencedaily.com/releases/2013/07/130715091222.htm.

[113] Carrasco M., Horiszny J. (2024). A Fatal Case of Powassan Virus Encephalitis. J. Brown Hosp. Med..

[114] New York State Department of Health Powassan (POW) Virus Disease Fact Sheet. https://www.health.ny.gov/diseases/communicable/powassan/.

[115] Hills S.L., Poehling K.A., Chen W.H., Staples J.E. (2023). Tick-Borne Encephalitis Vaccine: Recommendations of the Advisory Committee on Immunization Practices, United States, 2023. Morb. Mortal. Wkly. Rep. Recomm. Rep..

[116] Sung S., Wurcel A.G., Whittier S., Kulas K., Kramer L.D., Flam R., Roberts J.K., Tsiouris S. (2013). Powassan meningoencephalitis, New York, New York, USA. Emerg. Infect. Dis..

[117] Yeung A.W.K., Torkamani A., Butte A.J., Glicksberg B.S., Schuller B., Rodriguez B., Ting D.S.W., Bates D., Schaden E., Peng H. (2023). The promise of digital healthcare technologies. Front. Public Health.

[118] Al-Tarbsheh A., Jain E., Austin A. (2022). Powassan virus encephalitis: Single center experience from capital district of New York. Am. J. Med. Sci..

[119] Lange R.E., Dupuis A.P., Prusinski M.A., Maffei J.G., Koetzner C.A., Ngo K., Backenson B., Oliver J., Vogels C.B.F., Grubaugh N.D. (2023). Identification and characterization of novel lineage 1 Powassan virus strains in New York state. Emerg. Microbes Infect..

[120] McDonald E., Martin S.W., Landry K., Gould C.V., Lehman J., Fischer M., Lindsey N.P. (2019). West Nile Virus and Other Domestic Nationally Notifiable Arboviral Diseases—United States, 2018. MMWR Morb. Mortal. Wkly. Rep..

[121] (2020). Backyard Bug Busters. NJ Man Dies from Powassan Virus. https://www.backyardbugbusters.com/news/resource_center_2020_nj_man_dies_from_powassan_virus.php.

[122] Cumbie A.N., Whitlow A.M., Eastwood G. (2022). First Evidence of Powassan Virus (*Flaviviridae*) in *Ixodes scapularis* in Appalachian Virginia, USA. Am. J. Trop. Med. Hug..

[123] Burakoff A., Lehman J., Fischer M., Staples J.E., Lindsey N.P. (2018). West Nile Virus and Other Nationally Notifiable Arboviral Diseases—United States, 2016. MMWR Morb. Mortal. Wkly. Rep..

[124] Vahey G.M., Mathis S., Martin S.W., Gould C.V., Staples J.E., Lindsey N.P. (2021). West Nile Virus and Other Domestic Nationally Notifiable Arboviral Diseases—United States, 2019. MMWR Morb. Mortal. Wkly. Rep..

[125] Indiana State Department of Health Powassan Virus. https://www.in.gov/health/idepd/zoonotic-and-vectorborne-epidemiology-entomology/zoonotic-diseases/powassan-virus/.

[126] Farrington M., Ginsberg M., Pangonis S.F., Elenz J., Chiu C.Y., Miller S. (2023). Powassan Virus Infection Detected by Metagenomic Next-Generation Sequencing, Ohio, USA. Emerg. Infect. Dis..

[127] (2025). Illinois Resident Diagnosed with Powassan Virus; IDPH Launches Statewide Tick Testing. https://www.nbcchicago.com/news/local/illinois-resident-sickened-by-tick-borne-illness-never-reported-in-state-before/3828093/.

[128] Patel K.M., Johnson J., Zacharioudakis I.M., Boxerman J.L., Flanigan T.P., Reece R.M. (2018). First confirmed case of Powassan neuroinvasive disease in Rhode Island. IDCases.

[129] Castro A. Second Powassan Case of 2026 Confirmed; RI Health Officials Urge Precautions to Reduce Tick Exposure. *Rhode Island Current*, 13 August 2026. https://rhodeislandcurrent.com/2026/08/13/second-powassan-case-of-2026-confirmed-ri-health-officials-urge-precautions-to-reduce-tick-exposure/.

[130] Rhode Island Department of Health RIDOH Confirms a Case of Rare Tick-Borne Viral Infection (Powassan). https://www.ri.gov/press/view/47192.

[131] Bay to Bay News DNREC Reports New, Potentially Fatal Tick-Borne Illness in Delaware. https://baytobaynews.com/stories/dnrec-reports-new-potentially-fatal-tick-borne-illness-in-delaware,293078.

[132] De la Fuente J., Estrada-Peña A., Rafael M., Almazán C., Bermúdez S., Abdelbaset A.E., Kasaija P.D., Kabi F., Akande F.A., Ajagbe D.O. (2023). Perception of Ticks and Tick-Borne Diseases Worldwide. Pathogens.

[133] Colorado Tick-Borne Disease Awareness Association (COTBDAA) Powassan Virus. https://coloradoticks.org/what-is-powassan-virus/.

[134] Centers for Disease Control and Prevention Data and Maps for Powassan. https://www.cdc.gov/powassan/data-maps/index.html.

[135] Borham A., Motaal K.A., ElSersawy N., Ahmed Y.F., Mahmoud S., Musaibah A.S., Abdelnaser A. (2025). Climate change and zoonotic disease outbreaks: Emerging evidence from epidemiology and toxicology. Int. J. Environ. Res. Public Health.

[136] WHO/FAO/OIE/UNEP (2019). Taking a Multisectoral, One Health Approach: A Tripartite Guide to Addressing Zoonotic Diseases in Countries.

[137] Yale School of Public Health (2023). Tick-Borne Powassan Virus Is Transmitted in Concentrated Clusters in New England. https://ysph.yale.edu/news-article/tick-borne-powassan-virus-is-being-transmitted-in-concentrated-clusters-in-new-england-yale-study-says/.

[138] Ebel G.D., Foppa I., Spielman A., Telford S.R. (1999). A focus of deer tick virus transmission in the northcentral United States. Emerg. Infect. Dis..

[139] El Khoury M.Y., Hull R.C., Bryant P.W., Escuyer K.L., George K.S., Wong S.J., Nagaraja A., Kramer L., Dupuis A.P., Purohit T. (2013). Diagnosis of acute deer tick virus encephalitis. Clin. Infect. Dis..

[140] Centers for Disease Control and Prevention Current Year Data on Powassan Virus. https://www.cdc.gov/powassan/data-maps/current-year-data.html.

[141] Fagre A.C., Lyons S., Staples J.E., Lindsey N. (2023). West Nile Virus and Other Nationally Notifiable Arboviral Diseases—United States, 2021. MMWR Morb. Mortal. Wkly. Rep..

[142] Padda H., Jacobs D., Gould C.V., Sutter R., Lehman J., Staples J.E., Lyons S. (2025). West Nile Virus and Other Nationally Notifiable Arboviral Diseases—United States, 2023. MMWR Morb. Mortal. Wkly. Rep..

[143] WSAW News (2025). DHS Recommends Testing After Death in Wisconsin.

[144] New Jersey Department of Health, Communicable Disease Service Powassan Virus. https://www.nj.gov/health/cd/topics/powassan.shtml.

[145] Vahey G.M., Wilson N., McDonald E., Fitzpatrick K., Lehman J., Clark S., Lindell K., Pastula D.M., Perez S., Rhodes H. (2022). Seroprevalence of Powassan Virus Infection in an Area Experiencing a Cluster of Disease Cases: Sussex County, New Jersey, 2019. Open Forum Infect. Dis..

[146] Centers for Disease Control and Prevention (2019). Surveillance Guide for Reporting Arboviral Diseases—Human Case Data Only—Version 1.1.

[147] Cavanaugh C.E., Muscat P.L., Telford S.R., Goethert H., Pendlebury W., Elias S.P., Robich R., Welch M., Lubelczyk C.B., Smith R.P. (2017). Fatal Deer Tick Virus Infection in Maine. Clin. Infect. Dis..

[148] Centers for Disease Control and Prevention (2024). Guidelines for West Nile Virus Surveillance and Control.

[149] Kekatos M. Connecticut Woman Dies From Rare Tick-Borne Virus in 2nd Fatality This Year in US. *ABC News*, 10 June 2022. https://abcnews.com/Health/connecticut-woman-dies-rare-tickborne-virus-2nd-fatality/story?id=85308162.

[150] Fox News Rare Tick-Borne Virus Diagnosed in Northeastern US State. https://www.foxnews.com/health/rare-tick-borne-virus-causing-neurological-symptoms-diagnosed-northeastern-state.

[151] Centers for Disease Control and Prevention (2026). ArboNET.

[152] Maine CDC Identifies First West Nile and Powassan Virus Cases of the Season. https://www.maine.gov/dhhs/news/maine-cdc-identifies-first-west-nile-and-powassan-virus-cases-season-tue-07292025-1200.

[153] Maine Center for Disease Control and Prevention (2026). Human Powassan Case in Maine.

[154] WBAY News One Dead from Tickborne Powassan Virus; State Health Officials Urge Early Testing. https://www.wbay.com/2025/06/25/one-dead-tickborne-powassan-virus-state-health-officials-urge-early-testing/.

[155] Centers for Disease Control and Prevention (2026). Current Year Data (2026): Eastern Equine Encephalitis Virus.

[156] Eisen R.J., Paddock C.D. (2021). Tick and Tickborne Pathogen Surveillance as a Public Health Tool in the United States. J. Med. Entomol..

[157] Eisen R.J., Eisen L. (2018). The Blacklegged Tick, *Ixodes scapularis*: An Increasing Public Health Concern. Trends Parasitol..

[158] Eisen R.J., Kugeler K.J., Eisen L., Beard C.B., Paddock C.D. (2017). Tick-Borne Zoonoses in the United States: Persistent and emerging threats to human health. Ilar J..

[159] Beard C.B., Eisen L., Eisen R.J. (2021). The Rise of Ticks and Tickborne Diseases in the United States-Introduction. J. Med. Entomol..

[160] Maine Center for Disease Control and Prevention, Division of Disease Surveillance (2024). Lyme and Other Tickborne Illnesses 2023 Annual Report.

[161] Hadler J.L., Patel D., Nasci R.S., Petersen L.R., Hughes J.M., Bradley K., Etkind P., Kan L., Engel J. (2015). Assessment of Arbovirus Surveillance 13 Years after Introduction of West Nile Virus, United States. Emerg. Infect. Dis..

[162] Spectrum News Maine First Maine Death from Tick-Borne Powassan Virus Reported in 2024. https://spectrumlocalnews.com/me/maine/health/2024/06/13/first-maine-death-from-tick-borne-powassan-virus-reported-in-2024.

[163] Town of Sharon, MA Tick-Borne Powassan Virus Reported in Sharon. https://www.wbur.org/news/2024/04/29/powassan-ticks-virus-sharon-massachusetts.

[164] Centers for Disease Control and Prevention Oropouche in the United States. https://www.cdc.gov/oropouche/data-maps/current-year-data.html.

[165] Barboza P., Vaillant L., Le Strat Y., Hartley D.M., Nelson N.P., Mawudeku A., Madoff L.C., Linge J.P., Collier N., Brownstein J.S. (2014). Factors influencing performance of internet-based biosurveillance systems used in epidemic intelligence for early detection of infectious diseases outbreaks. PLoS ONE.

[166] Brackney D.E., Vogels C.B.F. (2023). The known unknowns of Powassan virus ecology. J. Med. Entomol..

[167] Chan E.H., Brewer T.F., Madoff L.C., Pollack M.P., Sonricker A.L., Keller M., Freifeld C.C., Blench M., Mawudeku A., Brownstein J.S. (2010). Global capacity for emerging infectious disease detection. Proc. Natl. Acad. Sci. USA.

[168] Madoff L.C. (2004). ProMED-mail: An early warning system for emerging diseases. Clin. Infect. Dis..

[169] Sutter R.A., Lyons S., Gould C.V., Staples J.E., Lindsey N.P. (2024). West Nile Virus and Other Nationally Notifiable Arboviral Diseases—United States, 2022. MMWR Morb. Mortal. Wkly. Rep..

[170] Meckawy R., Stuckler D., Mehta A., Al-Ahdal T., Doebbeling B.N. (2022). Effectiveness of early warning systems in the detection of infectious diseases outbreaks: A systematic review. BMC Public Health.

[171] Brownstein J.S., Freifeld C.C., Madoff L.C. (2009). Digital disease detection--harnessing the Web for public health surveillance. N. Engl. J. Med..

[172] Tavakoli N.P., Wang H., Dupuis M., Hull R., Ebel G.D., Gilmore E.J., Faust P.L. (2009). Fatal case of deer tick virus encephalitis. N. Engl. J. Med..

[173] Curren E.J., Lehman J., Kolsin J., Walker W.L., Martin S.W., Staples J.E., Hills S.L., Gould C.V., Rabe I.B., Fischer M. (2018). West Nile Virus and Other Nationally Notifiable Arboviral Diseases—United States, 2017. MMWR Morb. Mortal. Wkly. Rep..

[174] Kaur M., Adam M., Mladinich M.C. (2025). Pathogenicity and virulence of Powassan virus. Virulence.

[175] Allgaier J., Quarles R., Skiest D. (2019). Possible Prognostic Value of Serial Brain MRIs in Powassan Virus Encephalitis. Emerg. Infect. Dis..

[176] Taylor L., Condon T., Destrampe E.M., A Brown J., McGavic J., Gould C.V., Chambers T.V., I Kosoy O., Burkhalter K.L., Annambhotla P. (2021). Powassan virus infection likely acquired through blood transfusion. Clin. Infect. Dis..

[177] Powassan Virus Encephalitis—USA: (WI) Transfusion. 2020; 20200616.7473879. https://promedmail.org/promed-post/?id=20200616.7473879.

[178] Powassan Virus: Does It Always Cause Encephalitis?. https://danielcameronmd.com/no-neurologic-damage-three-children-lyme-disease-powassan-virus/.

[179] Blatman Z., Rowan-Legg A., Schaffzin J.K., Wilson N., Bechard N. (2024). Powassan virus encephalitis in a 9-year-old. CMAJ.

[180] People Magazine Infant Contracted Powassan Virus from Tick Bite in Martha’s Vineyard. https://people.com/powassan-infant-tick-bite-marthas-vineyard-virus-newborn-11786234.

[181] Taylor-Salmon E., Shapiro E.D. (2024). Tick-borne infections in children in North America. Curr. Opin. Pediatr..

[182] Sullivan M.D., Glose K., Sward D. (2024). Tick-Borne Illnesses in Emergency and Wilderness Medicine. Emerg. Med. Clin. N. Am..

[183] Wilson N., Vahey G.M., McDonald E., Fitzpatrick K., Lehman J., Clark S., Lindell K., Pastula D.M., Perez S., Rhodes H. (2023). Tick bite risk factors and prevention measures in an area with emerging Powassan virus disease. Public Health Chall..

[184] Global Lyme Alliance Most Common Tick-Borne Diseases. https://www.globallymealliance.org/about-lyme/prevention/other-tick-borne-diseases/.

[185] Hcini N., Lambert V., Picone O., Carod J.-F., Carles G., Pomar L., Epelboin L., Nacher M. (2024). Arboviruses and pregnancy: Are the threats visible or hidden?. Trop. Dis. Travel Med. Vaccines.

[186] Kapoor T., Murray L., Kuvaldina M., Jiang C.S., Peace A.A., Agudelo M., Jurado A., Robbiani D.F., Klemens O., Lattwein E. (2024). Prevalence of Powassan Virus Seropositivity Among People with History of Lyme Disease and Non-Lyme Community Controls in the Northeastern United States. Vector Borne Zoonotic Dis..

[187] Yale New Haven Health (2022). Spot the Symptoms of Powassan Virus, Lyme and Other Tick-Borne Illnesses.

[188] Romero J.R., Simonsen K.A. (2008). Powassan encephalitis and Colorado tick fever. Infect. Dis. Clin. N. Am..

[189] Steere A.C., Strle F., Wormser G.P., Hu L.T., Branda J.A., Hovius J.W., Li X., Mead P.S. (2016). Lyme borreliosis. Nat. Rev. Dis. Primers.

[190] Dumler J.S., Choi K.S., Garcia-Garcia J.C., Barat N.S., Scorpio D.G., Garyu J.W., Bakken J.S. (2005). Human granulocytic anaplasmosis. Emerg. Infect. Dis..

[191] Feder H.M., Telford S., Goethert H.K., Wormser G.P. (2021). Powassan Virus Encephalitis Following Brief Attachment of Connecticut Deer Ticks. Clin. Infect. Dis..

[192] Krause P.J., Narasimhan S., Wormser G.P., Rollend L., Fikrig E., Lepore T., Barbour A., Fish D. (2013). Human *Borrelia miyamotoi* infection. N. Engl. J. Med..

[193] Frost H.M., Schotthoefer A.M., Thomm A.M., Dupuis A.P., Kehl S.C., Kramer L.D., Fritsche T.R., Harrington Y.A., Knox K.K. (2017). Serologic Evidence of Powassan Virus Infection in Patients with Suspected Lyme Disease. Emerg. Infect. Dis..

[194] Tokarz R., Tagliafierro T., Cucura D.M., Rochlin I., Sameroff S., Lipkin W.I. (2017). Detection of *Anaplasma phagocytophilum*, *Babesia microti*, *Borrelia burgdorferi*, *Borrelia miyamotoi*, and Powassan Virus in Ticks by a Multiplex Real-Time Reverse Transcription-PCR Assay. mSphere.

[195] Aliota M.T., Dupuis A.P., Wilczek M.P., Peters R.J., Ostfeld R.S., Kramer L.D. (2014). The prevalence of zoonotic tick-borne pathogens in *Ixodes scapularis* collected in the Hudson Valley, New York State. Vector Borne Zoonotic Dis..

[196] Knox K.K., Thomm A.M., Harrington Y.A., Ketter E., Patitucci J.M., Carrigan D.R. (2017). Powassan/Deer Tick Virus and *Borrelia* Burgdorferi Infection in Wisconsin Tick Populations. Vector Borne Zoonotic Dis..

[197] Yuan Q., Llanos-Soto S.G., Gangloff-Kaufmann J.L., Lampman J.M., Frye M.J., Benedict M.C., Tallmadge R.L., Mitchell P.K., Anderson R.R., Cronk B.D. (2020). Active surveillance of pathogens from ticks collected in New York State suburban parks and schoolyards. Zoonoses Public Health.

[198] Rincón T.C., Kapoor T., Keeffe J.R., Simonelli L., Hoffmann H.-H., Agudelo M., Jurado A., Peace A., Lee Y.E., Gazumyan A. (2024). Human antibodies in Mexico and Brazil neutralizing tick-borne flaviviruses. Cell Rep..

[199] Stone E.T., Hassert M., Geerling E., Wagner C., Brien J.D., Ebel G.D., Pinto A.K. (2022). Balanced T and B cell responses for immune protection against Powassan virus. Cell Rep..

[200] Osmosis/Elsevier (2025). Powassan Virus: What Is It, Causes, Treatment, and More.

[201] ICliniq How to Manage Complications of Powassan Virus Infection. https://www.icliniq.com/articles/infectious-diseases/management-of-powassan-virus-complications.

[202] Vermont Department of Health Powassan Virus. https://www.healthvermont.gov/disease-control/tick-bite-illnesses/powassan-virus.

[203] Ellwanger J.H., da Veiga A.B.G., Kaminski V.D.L., Valverde-Villegas J.M., de Freitas A.W.Q., Chies J.A.B. (2021). Control and prevention of infectious diseases from a One Health perspective. Genet. Mol. Biol..

[204] Ghai R.R., Wallace R.M., Kile J.C., Shoemaker T.R., Vieira A.R., Negron M.E., Shadomy S.V., Sinclair J.R., Goryoka G.W., Salyer S.J. (2022). A generalizable one health framework for the control of zoonotic diseases. Sci. Rep..

[205] Li T., Zhou X.-N., Tanner M. (2025). One Health: Enabler of effective prevention, control and elimination of emerging and re-emerging infectious diseases. Infect. Dis. Poverty.

[206] Massachusetts Department of Public Health (2024). Powassan Virus.

